# Point-of-care mass spectrometry metabolomic analysis enabling intraoperative brain tumor diagnosis

**DOI:** 10.7150/thno.113336

**Published:** 2025-07-24

**Authors:** Junhan Wu, Xinqi Fang, Haoyue Zhang, Hao Xu, Peter Jih Cheng Wong, Ying Mao, Wenpeng Zhang, Zheng Ouyang, Wei Hua

**Affiliations:** 1 State Key Laboratory of Precision Measurement Technology and Instruments, Department of Precision Instrument, Tsinghua University, Beijing 100084, China.; 2 Department of Neurosurgery, Huashan Hospital, Fudan University, Shanghai, 200040, China.; 3 National Center for Neurological Disorders, Shanghai 200040, China.; 4 Shanghai Key Laboratory of Brain Function Restoration and Neural Regeneration, Shanghai 200040, China.; 5 Institute of Neurosurgery, Fudan University, Shanghai 200040, China.; 6 Shanghai Clinical Medical Center of Neurosurgery, Shanghai 200040, China.

**Keywords:** miniature mass spectrometer, point-of-care testing, metabolomic, intraoperative analysis, brain tumor

## Abstract

**Rationale:** While mass spectrometry (MS) is known for being capable of analyzing a wide range of biomarkers, its usages in clinical settings have been hindered by the stringent requirements for operating the MS analysis system as well as performing the analytical procedure at the point of care (POC). We have developed a miniature MS system and extremely simplified analytical protocols for POC analysis of tumors. It enabled comprehensive metabolite profiling with brain tissue biopsy, which allowed accurate and real-time diagnosis of brain tumors and guiding of surgical resection strategy.

**Methods:** A miniature linear ion trap MS system with direct sampling ionization for tissue biopsy analysis was developed, which is suitable for performing real-time analysis in a surgical room. A segment scan method was developed to allow coverage of a wide range of metabolites while providing adequate sensitivity. An extremely simplified protocol was developed to allow a real-time analysis of the tissue samples taken from patients. The development and validation of this method involved 137 brain tissue samples from 109 patients. The miniature MS system could operate on battery without requiring compressed gas or large amounts of solvents like traditional MS analysis systems in lab.

**Results:** With optimized sampling and scanning method, 183 metabolites in the range of m/z 50 - 500 could be identified from human brain tissues after a 2 min analysis time. Metabolomic features were extracted that allowed distinctions between glioma and normal tissues (at an area under the curve (AUC) of 0.932), isocitrate dehydrogenase (IDH) mutant and wildtype gliomas (at an AUC of 1.000), and IDH mutant gliomas and glioblastoma (at an AUC of 1.000). A comparison study was also carried out between fresh and stored tissue samples and decrease in abundance was observed for metabolites such as glutamate and N-acetylaspartic acid, which indicates the importance of real-time analysis of the biological samples for clinical applications.

**Conclusions:** This study shows the potential of performing intraoperative or bedside MS analysis of a panel of metabolomic biomarkers in real time to facilitate the diagnosis of the disease as well as the decision making for surgery or therapy. Miniature mass spectrometry system with direct sampling ionization method serves as a valid platform for transferring MS analysis from traditional laboratories to clinical settings.

## Introduction

The significance for using molecular biomarkers for disease diagnosis and evaluation for therapy has long been recognized^1^. Mass spectrometry is suitable for analyzing of a wide variety of molecular biomarkers at high confidence for identification and high accuracy for quantitation^2^. Its use in clinical settings to support real-time diagnosis or decision making, however, is hindered by a series of problems related to the stringent requirements for both maintaining the analytical system as well as performing the analysis. There has been a major effort in developing MS analysis systems for point-of-care (POC) applications^2^, ^3^, with a recent effort in assisting real-time decision making for brain tumor surgery^4^. Taking glioma as an example, there is a variety of mutations in oncogenes and tumor suppressor genes that lead to the development and progression of brain tumors^5^, such as IDH1/IDH2 mutations, 1p/19q co-deletion, telomerase reverse transcriptase (TERT) promoter mutations, and histone H3 alterations. It is highly desirable to obtain accurate information on the tumor type to facilitate the determination of the surgery strategy, which has always been a delicate balance between the maximal removal of the tumor tissue and the preservation of the brain functions. For example, gliomas with IDH mutations show better prognosis and the patients would benefit from adjuvant therapy and extensive neurosurgical resection^6^, ^7^.

For glioma classification, gene sequencing has been routinely used to provide accurate information, but it is not possible to perform on-site for surgery. Immunochemical staining is another method widely used, detecting the protein markers in tissues, but also is time-consuming and has limited applicability^8^. These methods serve well as postoperative evaluation but are not feasible for supporting real-time decision on resection strategy. A major effort has also been put in discovering proteomic^9^, ^10^ and metabolomic^11^ hallmarks for glioma classification. Conventional MS analysis using liquid chromatography mass spectrometry (LC-MS) is not feasible for POC analysis either, due to the strict requirements for installing and maintaining the systems as well as the complex procedures for the analysis. Recently advancement for miniature mass spectrometry systems with direct analysis capability sheds light on the transfer of mass spectrometry from traditional analytical laboratories to the clinical settings for on-site clinical sample analysis and real-time decision making.

The development of POC MS systems has gone through a long journey^2^, ^3^, ^12^ with some major breakthroughs made for miniaturization of the mass spectrometer, improvement to the adaptability to clinical environment, and simplification in sample treatment. Shoebox-size MS systems become available that is capable of analyzing non-volatile biological compounds from clinical samples, retaining MS/MS capability that is critical for analyzing target biomarkers from complex samples at high confidence and high sensitivity^13^-^15^. The direct analysis capability advanced significantly with the emerging of ambient ionization^16^, ^17^, which aimed at ionizing and analyzing complex samples with minimal pre-treatment. Examples for clinical application include Mass Spec Pen^18^ and rapid evaporative ionization^19^, which were designed for intraoperative analysis but currently still used with large lab-type mass spectrometers. Methods such as paper spray ionization^20^, ^21^ or paper capillary spray^22^ were used for developing disposable sample cartridges for use with miniature mass spectrometry systems. Recently, IDH1/IDH2 glioma diagnoses could be performed intraoperatively with biopsy tissue samples using the oncometabolite 2-hydroxyglutarate (2-HG)^4^, which alters epigenetic regulation and contributes to tumor progression.

While IDH1/IDH2 gliomas could be readily identified with 2-HG^23^, other types of gliomas might not be diagnosed with MS analysis of a single-compound biomarkers, which also applies for most of the cases for cancer diseases. Omics panels with multiple relevant targets could possibly serve as effective biomarkers^24^, ^25^. While protein analysis still requires complex sample treatment prior to mass spectrometry, fast extraction of metabolites from raw tissue samples could be effectively achieved by direct sampling techniques^18^, ^19^, ^26^. Analyzing metabolite compounds of a wide variety, within a relatively wide mass range and over a wide dynamic concentration range still represents a significant challenge for POC analysis using miniature MS systems without sophisticated sample purification and chromatographic separation. This calls for further development in instrumentation and analytical methods and careful validations for clinical applications.

In this work, we developed a POC metabolomic characterization method using a novel miniature MS system with direct sampling ionization for on-site analysis of biopsy tissue samples in a surgery room to support real-time decision making. A segment scan method was developed for the ion trap mass spectrometer to enable scanning of more than 180 metabolites at a wide m/z range while providing adequate sensitivity. An extremely simplified protocol was developed for POC MS analysis of tissue samples taken from patients. A cohort of 137 human brain tissue samples from 109 patients was used for the development and validation of the method, including gliomas with IDH mutation and wildtype, normal brain tissues and different subtypes of gliomas such as astrocytoma, oligodendroglioma and glioblastoma. With many metabolite features extracted from MS analysis, distinguishing different types of brain tumors was achieved. With the capability of POC metabolomic analysis, the miniature MS system has the potential to be further used for intraoperative diagnosis of different types of cancers.

## Results

### Development of POC MS Method for Rapid Profiling of Metabolites

**Figure 1:**
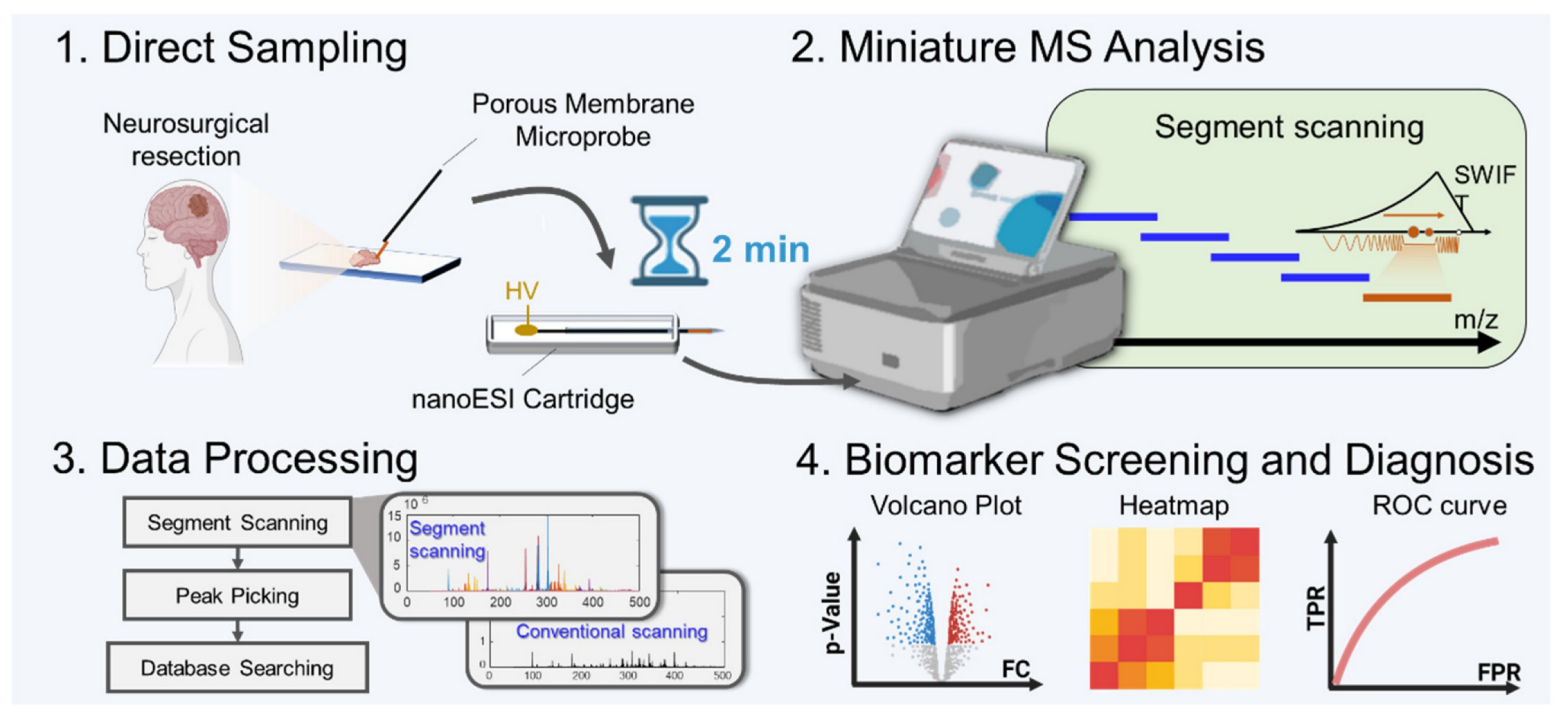
Workflow of detection of brain tumors based on comprehensive profiling of metabolites by the miniature MS system.

A novel miniature MS system designed for POC analysis^4^, ^27^ was further developed for metabolomic characterization (Figure 1). It is of small size and can operate on battery without consuming large amounts of gas or solvents, which are typically required for conventional MS systems. To simplify the analytical procedure, a microprobe was developed for direct sampling of metabolites from biopsy tissue samples. It consisted of a stainless-steel wire, with a coating of porous polypropylene membrane of 100-μm thickness. During sampling, the microprobe was rolling on the surface of the fresh tissue for sampling of the whole metabolome from the tissue. After sampling, it was inserted into a nanoESI emitter containing 10 μL of solvent (ethanol/H_2_O, 9/1, v/v) for direct ionization and fast MS analysis. The ionization remained stable for more than 1 min, which can provide adequate time widow for profiling of hundreds of metabolites based on MS or MS/MS experiments.

**Figure 2:**
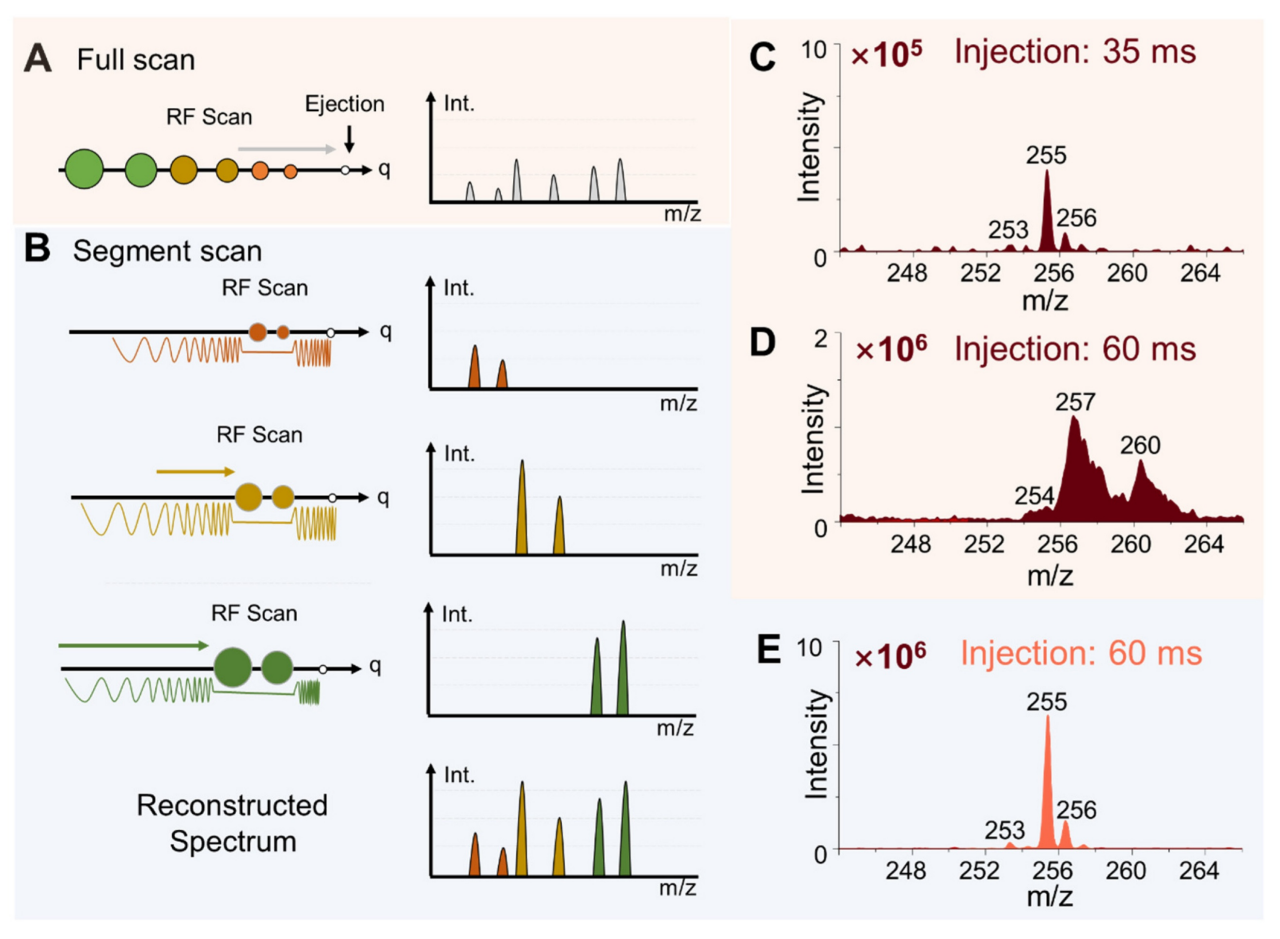
Schematics of the (A) conventional full scan method and (B) segment scan method. Comparison of mass spectra obtained using full scan method with injection time of (C) 35 ms and (D) 60 ms. (E) Mass spectrum obtained by the segment scan method with injection time of 60 ms.

The miniature MS system was designed with a configuration using a linear ion trap (LIT) as the mass analyzer and a discontinuous atmospheric pressure interface (DAPI)^13^ for ion introduction (Figure S1), which is essential for allowing the miniaturization of the MS system as well as reserving the MS/MS capability^28^. However, the analysis performance of ion trap instruments is known for being vulnerable to space charge effect, which limits the dynamic range and sensitivity for omics analysis that needs to cover a relatively large number of analytes in a wide range of concentration. Although the direct sampling method simplifies the sample preparation for MS analysis, the inevitable matrix effects increase the likelihood of space charge effects. To overcome this problem, an ion number control mechanism was first implemented. After the DAPI was opened, the RF (radio frequency) applied on the LIT for trapping the ions introduced was turned after a delay so the total number of ions trapped in the LIT could be adjusted. The actual ion injection time is the overlap between the onset of the trapping RF and the offset of the DAPI. A segment scan method (Figure 2A, B) was also developed to further improve the performance of the system. As shown with the comparison of normal full scans with injection time of 35 ms and 60 ms for analyzing rat brain tissue samples (Figure 2C, D), significant space charge effects were overserved for injection time of 60 ms, leading to severely compromised resolution as well as suppression of the low-abundant peaks (Figure 2D). The increase in peak intensity was not be directly proportional to the injection time (Figure S2). The peaks at m/z 253 ([M-H]^-^ of FA 16:1), m/z 255 ([M-H]^-^ of FA 16:0) and m/z 256 (isotope of [M-H]^-^ of FA 16:0) exhibited significant broadening. In addition, peak shifts were observed, such as m/z 255 shifting to m/z 257 (Figure 2D), which significantly reduced the accuracy of the mass measurements.

The segment scan method was therefore implemented to address this problem, whereby ions in narrowed m/z ranges (20, 30, 40, 50, 70 and 100 Da) were separately analyzed and a full spectrum was reconstructed subsequently (Figure 2B). SWIFT (Stored waveform inverse Fourier transform) was used for isolating the ions in a narrow m/z window and the trapping RF voltage level was adjusted for selecting the m/z window. As shown in Figure 2E for a spectrum recorded for m/z 20-50 window, ions introduced at an injection time of 60 ms could now be analyzed with improved spectral intensity and a resolution of 0.4 Da FWHM (full width at half maximum) while remaining a unit resolution or better (Figure 2E). No peak shifts were observed at ion injection time of 60 ms or longer (Figure S2).

### Wide Coverage of Metabolites in Brain Tissue at High Sensitivity

**Figure 3:**
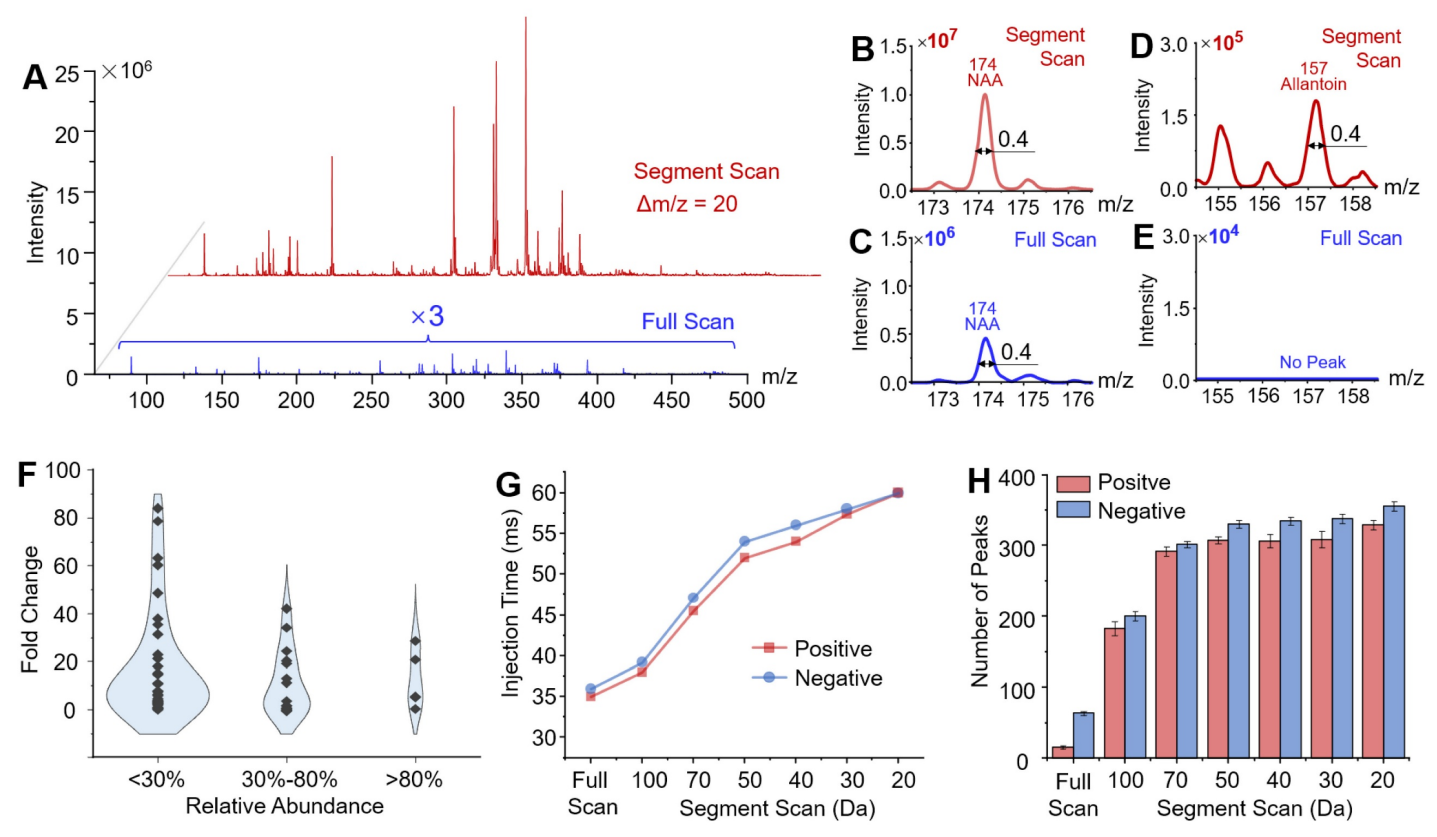
Development and optimization of segment scan mode. (A) Comparison of mass spectra of rat brain metabolites obtained by conventional full scan method and segment scan method at 20 Da window width. (B)-(E) Comparison of mass spectra at the range of m/z 172-177 and m/z 154-159. (F) Increase of identified peak numbers at different intensity ranges with the segment scan method in comparison with conventional full scan method. (G) Control of Average injection time for segment scan method. (H) The number of identified peaks by different scanning methods.

To facilitate comprehensive metabolomic profiling and maximize coverage of potential biomarkers, a workflow was developed using the miniature MS system. Segment windows were selected based on the mass ranges of the target metabolites. Corresponding SWIFT waveforms were generated for each m/z segment, and the proper ion injection times were also determined for the samples (Figure S3). The effectiveness was validated using rat brain tissue samples. Mass spectra of main metabolites were obtained in the m/z range of 65 - 500 in negative ion mode using the segment scan method, with each 20 Da segment window overlapping by 5 Da with adjacent segments. As shown in Figure 3A, the intensity of the mass spectral signal obtained by the segment scan method was significantly enhanced in comparison with the conventional by full scan method. For example, the intensity of N-acetylaspartic acid (NAA, [M-H]^-^ at m/z 174) increased by 20 folds using the segment scan method (Figure 3B and C). The peak of allantoin was not detected using the full scan method, while it was well detected at m/z 157 ([M-H]^-^) with the intensity of 1.6 × 10^5^ when using the segment scan method (Figure 3D-E). The segment scan method also significantly enhanced the detection of low-abundance peaks. As shown in Figure 3F, high-abundance peaks (intensity over 80% of the highest peak) showed an intensity increase of up to 29 times, while those with intensities between 30%-80% increased by up to 42 times. A much greater enhancement was observed for low-abundance peaks (intensity below 30% of the highest peak), with a maximum intensity increase of about 80 times. Moreover, narrowing the m/z acquisition window allowed more ions to be introduced into the ion trap at relatively high resolution (FWHM = 0.4 Da, Figure 3G). The increase in the signal intensity by the segment scan method was also validated in the analysis of lipids in tissues and metabolites in whole blood samples (Figure S4 and S5).

The enhanced sensitivity of MS detection also expanded the coverage of metabolite profiling using the miniature MS system. As illustrated in Figure 3H, reducing the segment window for scanning significantly increased the number of detected metabolite peaks (defined as having an absolute intensity > 5000) in rat brain tissue samples. With a segment window of 20 Da, over 600 metabolite peaks were detected in rat brain tissues (329 in positive ion mode and 355 in negative ion mode), far exceeding the peak numbers detected by the conventional full scan method (15 in positive ion mode and 63 in negative ion mode). The detected peaks were identified by matching them with the HMDB database, using a mass deviation threshold of ± 0.2 Da. In total, 227 compounds were identified, including 177 in positive ion mode and 171 in negative ion mode, with 121 metabolites detected in both modes. These compounds included organic acids and their derivatives, lipids, nucleosides, benzenoids, and others (Figure S6). The improved metabolite coverage provides additional features to better differentiate between tissues. For example, using 207 metabolite features detected through direct sampling and miniature MS analysis with segment scan, it allows for clearly distinguishing the cerebrum and cerebellum regions in the rat brain tissue (Figure S7).

### Diagnosis of Gliomas and IDH Mutation Based on Metabolomic Profiling

**Figure 4:**
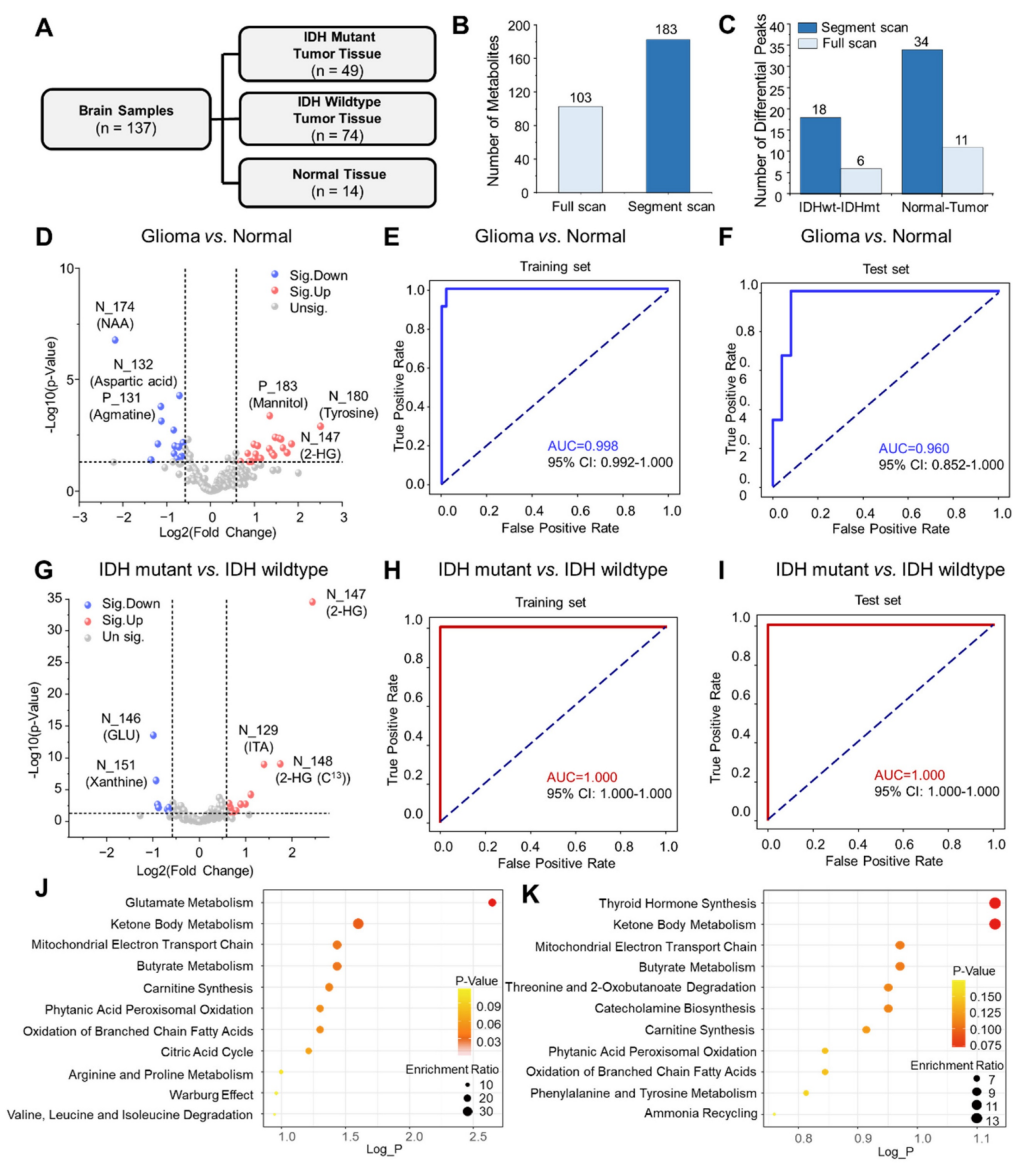
Detection of metabolites in glioma in brain tissue samples. (A) The composition of the cohort of brain tissues. (B) Number of metabolites identified from brain tissue samples by different scan methods. (C) Comparison of the number of differential metabolites by different scan methods. Volcanic plot and significantly differential metabolites that distinguish (D) glioma and normal tissues, and (G) IDH mutant and IDH wildtype gliomas using segment scan. ROC and AUC values for classification of (E) glioma versus normal tissues and (H) IDH mutant versus IDH wildtype gliomas with training set, and ROC of (F) glioma versus normal tissues and (I) IDH mutant versus IDH wildtype gliomas with training set using test set. Bubble plots of pathway enrichment in (J) glioma and normal tissues, and (K) IDH mutant and IDH wildtype gliomas.

The miniature MS system, combined with direct sampling and the segment scan method, was further applied to analyze metabolites in human brain tumor samples. A cohort of 137 human brain tissue samples from 109 patients was established, consisting of 49 IDH-mutant glioma samples, 74 IDH-wildtype glioma samples, and 14 normal brain tissue samples (Figure 4A). Through metabolite analysis by the miniature MS system, a total of 183 metabolites were consistently identified across the brain tissue samples (Figure 4B). The segment scan method significantly increased metabolite intensity, as well as the number of detected metabolite peaks in human brain tissues (Figure S8).

Volcano plot analysis was used to identify metabolite features that differentiate between tissue groups. A total of 34 metabolite features showed significant differences between glioma and normal human tissue samples (P < 0.05, fold change > 1.5 or < 2/3) (Figure 4C-D, Figure S9). The abundances of NAA ([M-H]^-^, m/z 174) and aspartic acid ([M-H]^-^, m/z 132) were observed to reduce significantly in glioma tissues compared to normal tissues, while the abundance of tyrosine ([M-H]^-^, m/z 180) increased significantly in glioma tissues. Using the miniature MS system, differential metabolite features were further used to distinguish different types of tissue. These features were used for further analysis by a receiver operating characteristic (ROC) curve, which achieved an area under the curve (AUC) of 0.982 (95% confidence interval: 0.992-1.000) for the training set and 0.929 for the test set, effectively distinguishing glioma from normal tissues (Figure 4E-F, Figure S11 and Table S4). The model achieved consistently high performance across all folds in the 5-fold cross-validation, indicating strong generalization ability and robustness (Figures S12-S13 and Table S5). For differentiating IDH-mutant from IDH-wildtype gliomas, volcano plot analysis identified 18 metabolite features with high confidence (P < 0.05, fold change > 1.5 or < 2/3) (Figure 4C, 4G). IDH-mutant glioma samples showed increased levels of 2-HG ([M-H]^-^, m/z 147) and decreased levels of glutamic acid ([M-H]^-^, m/z 146) and xanthine ([M-H]^-^, m/z 151) compared to wildtype gliomas (Figure 4G, Figure S9). These features were used to construct a ROC curve, achieving an AUC of 1.0 (95% confidence interval: 0.958-1.000) for distinguishing IDH-mutant gliomas in both training and test sets (Figure 4H-I). While the conventional scan method could also differentiate IDH-mutant from wildtype gliomas. The segment scan method demonstrated a lower P-value (5×10^-33^ vs. 4×10^-22^), indicating higher confidence for determination of IDH mutations (Figure S10).

Metabolic pathway enrichment analysis was performed with MetaboAnalyst based on differential metabolites identified above. As shown in Figure 4J, significant differences were observed in glutamate metabolism between IDH-mutant and wildtype gliomas. Additionally, ketone metabolism differed between tumor and normal tissues, as well as between IDH-mutant and wildtype gliomas (Figure 4K). These differences probably involve altered expression patterns of enzymes such as ACSS2 (acyl-CoA synthetase short-chain family member 2), BDH1 (3-hydroxybutyrate dehydrogenase type 1), and ACAT (acyl coenzyme A-cholesterol acyltransferase).

### Real-time Intraoperative Diagnosis and Metabolic Delineation

**Figure 5:**
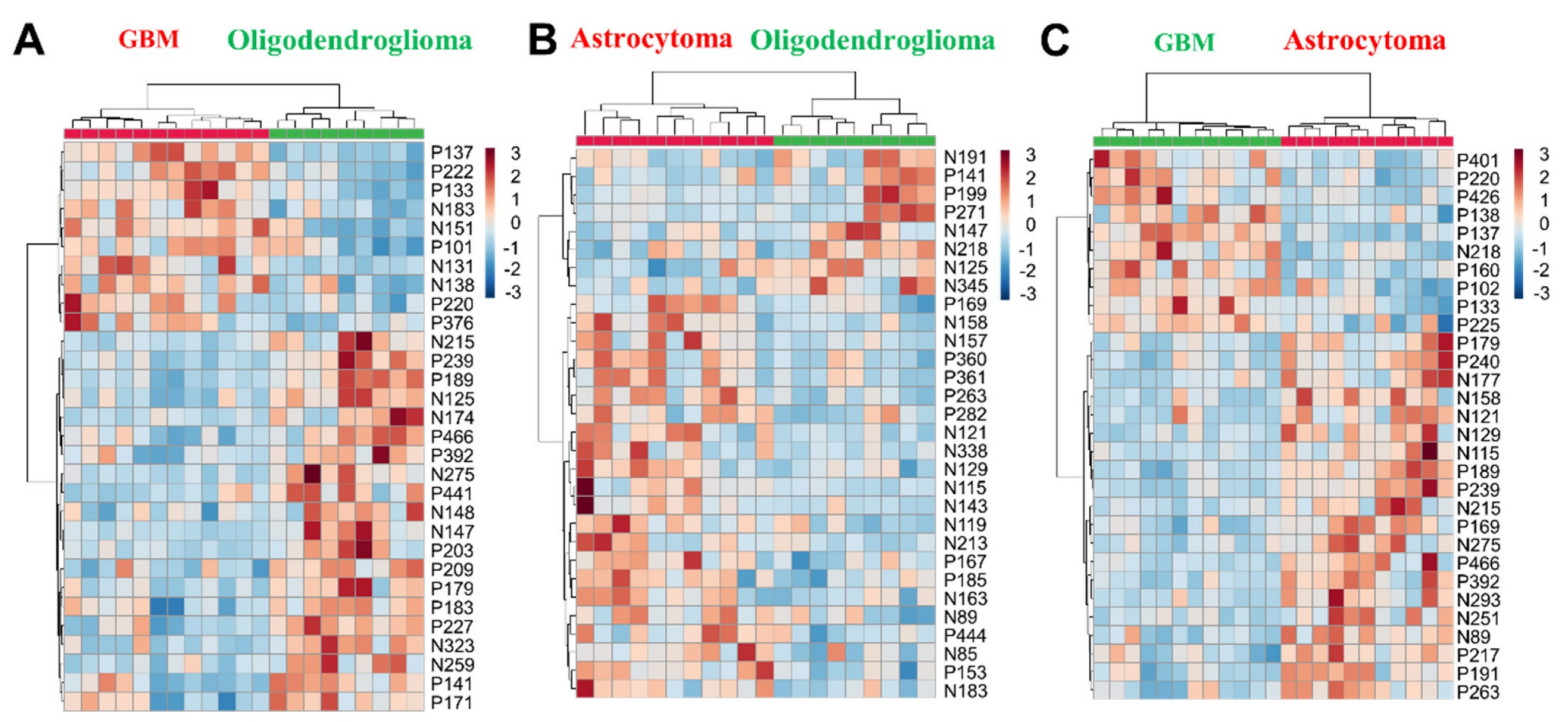
Hierarchical cluster analysis of (A) GBM vs. oligodendroglioma, (B) astrocytoma vs. oligodendroglioma, (C) GBM vs. astrocytoma using metabolomic features. Metabolomic feature notation: N for negative mode, P for positive mode, and the number for the m/z value (e.g., N146 indicates m/z 146 in negative mode).

The effectiveness of the miniature POC MS system for intraoperative diagnosis was demonstrated with an additional glioma cohort, including lower-grade gliomas (LGG, 20 biopsies: 11 astrocytomas and 9 oligodendrogliomas) and glioblastomas (GBM, 12 biopsies). Using segment scan with the miniature MS system, over 500 metabolic features were detected across an m/z range of 50 to 500. The top 30 differential metabolic features enabled clear intraoperative differentiation of lower-grade gliomas (sampling time: 2.0 ± 0.5 min) (Figure 5A-C, Figure S14 and Figure S15), with clustering results consistent with WHO CNS5 classification.

**Figure 6:**
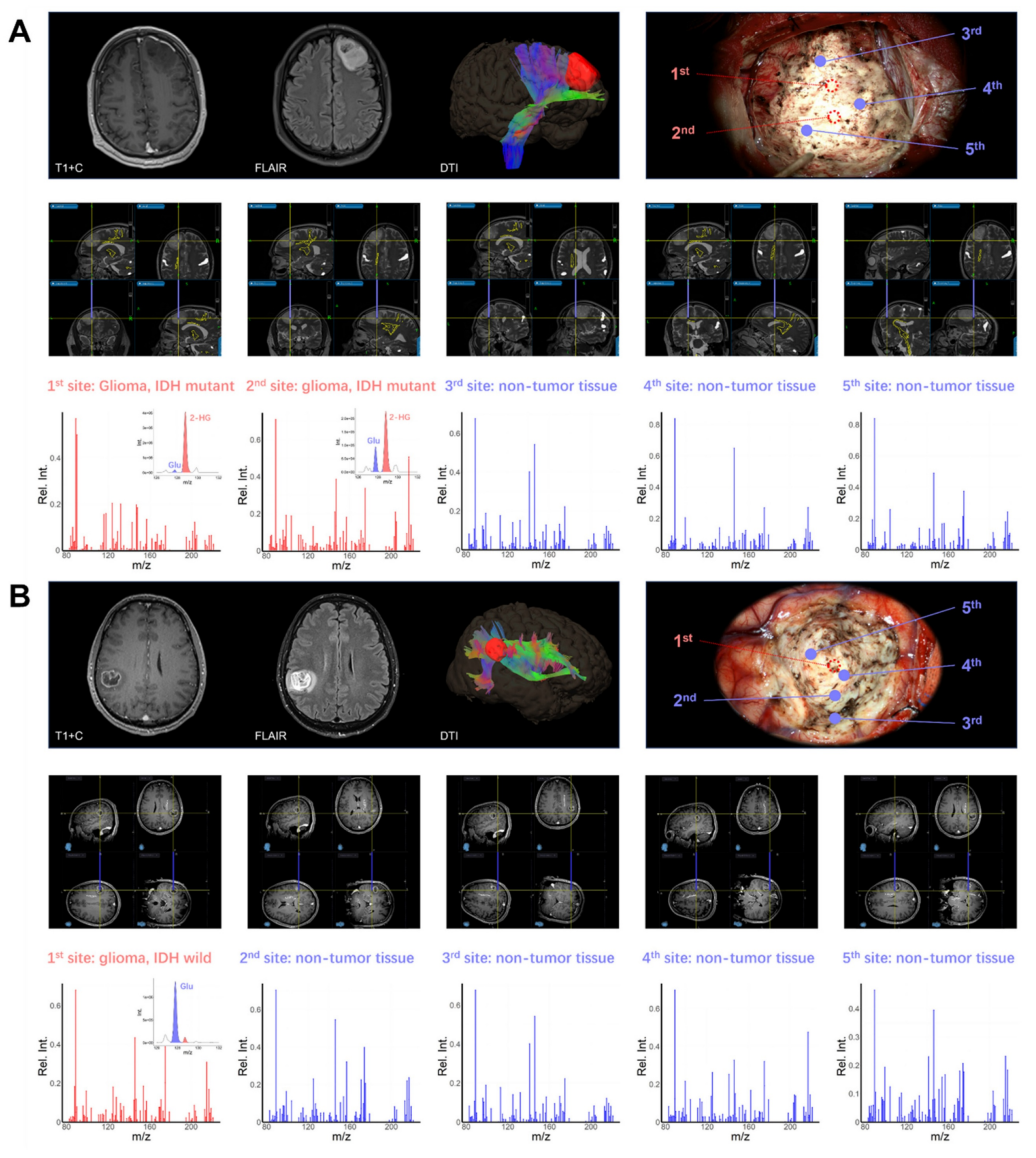
(A) Intraoperative metabolite analysis identified an IDH mutation in a left frontal lobe tumor, enabling precise metabolic boundary delineation for safe resection. (B) Intraoperative metabolite analysis differentiated glioma from normal tissue, aiding in the real-time intraoperative delineation of the tumor's edematous boundary.

To demonstrate the intraoperative diagnostic value of POC metabolomics, multi-site sampling and metabolic analysis were performed in six patients using intraoperative navigation. Two representative cases are demonstrated. In the first case, a left frontal glioma in a 45-year-old male was resected under microscopy with multi-modality neuro-navigation (Figure 6A). The classification model identified tumor core sites 1 and 2 as IDH-mutant glioma with confidence levels of 0.997 and 0.999, respectively. Normal sites 3, 4, and 5 were identified as non-glioma with confidence levels of 0.896, 0.910, and 0.898, indicating clear metabolic delineation. In the second case, a patient with a right parietal lobe tumor showed MRI enhancement one week prior to surgery (Figure 6B). According to intraoperative metabolomic analysis, tumor core site 1 was identified as IDH wildtype glioma with a confidence level of 0.999, while edema region sites 2 to 5 were identified as non-tumor tissue with confidence levels of 0.785, 0.810, 0.898, and 0.915, respectively. These results were validated through postoperative immunohistochemical testing (Figure S16). These results demonstrate the potential of intraoperative metabolomic analysis using a POC MS system to assist neurosurgeons in real-time tumor metabolic delineation and diagnosis.

## Discussion

In clinical diagnosis as well as therapy for diseases, it is highly desirable to have high confidence assessment based on molecular biomarkers. In brain tumor surgery, it is crucial to balance between brain function preservation with maximal resection^29^. Compared to traditional non-invasive imaging techniques, methods based on metabolic changes offer a more accurate molecular diagnosis and a deeper understanding of the nature of tumors^30^. This has driven the development of novel diagnostic techniques, such as positron emission tomography-computed tomography (PET-CT) for detecting glucose metabolism using fluorodeoxyglucose tracers^31^, ^32^, magnetic resonance spectroscopy for measuring metabolites such as 2-HG, choline, creatine, and N-acetyl aspartate^33^, and Raman spectroscopy for identifying molecules through vibrational modes^34^. Glioma subtypes exhibit distinct metabolic characteristics, with the accumulation of 2-HG in IDH-mutant gliomas^35^ and lipidomic alterations in GBM^36^, showing the importance and feasibility of metabolic characterization in surgery. MS is the gold standard for qualitative and quantitative analysis of a broad range of compounds. While traditional MS techniques such as LC-MS are highly sensitive for metabolite analysis, their complex preprocessing requirements and the need for specialized equipment make them challenging for routine clinical use.

In this work, we developed a novel miniature MS system capable of comprehensive metabolite profiling in tissue samples. By coupling direct sampling with segment scan, the miniature MS system effectively enhances the detection of low-abundance metabolites, significantly increasing the number of detectable metabolites from brain tissues. By overcoming the space charge effect, this method enables wide-range metabolomic data acquisition within a short timeframe. Comprehensive metabolic profiling can reveal new biomarkers for a wide range of diseases^37^, ^38^, allowing accurate diagnosis and classification of diseases^39^, ^40^. Our previous method successfully diagnosed IDH mutations using 2-HG/Glu ratios^4^, but differentiating other glioma subtypes still remained challenging due to the limitation in analyzing wide-range metabolites. The novel miniature MS approach enables scanning of more than 180 metabolites across a broad m/z range while providing adequate sensitivities, allowing identification of multiple glioma subtypes and showing potential for application to other brain tumors. It should be noted that the tumor samples were taken from the core of the tumor tissue; the presence of edema, necrosis, or infiltrative margins may influence the observed metabolic signatures.

Comprehensive metabolomic analysis has the potential to advance clinical research and improve neurosurgical outcomes. During brain tumor resection, metabolic profiling can assess the extent of residual tumor and any damage to surrounding healthy tissue^41^. This information assists neurosurgeons in evaluating surgical outcomes and making timely adjustments. Point-of-care metabolic analysis can also help predict complications, allowing preventive or therapeutic interventions. For instance, in cerebrovascular surgery, metabolic profiles can help predict brain edema or ischemia, enabling early intervention^42^. Besides, metabolic analysis provides insights into the patient's metabolic status, aiding in postoperative management. In brain hemorrhage surgery, shifts in metabolic compositions can reflect brain tissue recovery, helping guide and adjust postoperative rehabilitation efforts^43^.

The miniature MS system is capable of POC metabolite profiling, which also provides a unique advantage in identifying compounds prone to degradation during storage. For example, the abundances of metabolites in rat brain tissue, such as glutamic acid ([M-H]^-^, m/z 146) and N-acetyl aspartic acid ([M-H]^-^, m/z 174), were found to decrease gradually over seven days of frozen storage (Figure S17). Similarly, the abundance of choline ([M+H]^+^, m/z 104) also decreased, probably due to the hydrolysis of choline-containing compounds. A comparison was conducted between the analysis of metabolites in human glioma tissue immediately after resection in the operating room and after one month of frozen storage at -80 °C. Besides a reduction in glutamic acid and NAA and an increase in choline, significant changes were also observed in the abundances of dihydrouracil, tryptophan, lactic acid, and malic acid after storage (Figure S18). These findings suggest that metabolite profiling pf freshly resected samples could improve diagnostic accuracy for brain tumors.

In this work, we developed an intraoperative metabolic profiling workflow based on a miniature mass spectrometer and a direct sampling technique, demonstrating its potential for brain tumor classification. However, there are still some limitations that require further improvement. The use of an ion trap as the mass analyzer may not provide sufficient mass resolution to identify certain metabolites in brain tissue samples. This limitation has been partially addressed through MS/MS analysis, which offers characteristic product ions for metabolite identification. Besides, before clinical translation, external validation using larger sample cohorts would still be required. The reliability of the current machine learning model may be influenced by the limited number of patient samples. Due to the limited availability of tissue samples from brain tumor patients, we conducted internal validation to demonstrate the model's effectiveness. Further optimization may be possible with the inclusion of an external validation cohort.

In conclusion, we developed a miniature MS system with simplified protocols for POC analysis of metabolites in tissue samples. This approach enables accurate, real-time diagnosis of brain tumors and supports intraoperative decision-making for surgical resection. The implementation of a segment scan method provided broad metabolite coverage with sufficient sensitivity, while the highly simplified protocol enabled rapid analysis. The method was validated across both postoperative and intraoperative cohorts, including normal brain tissues, IDH-mutant, and IDH-wildtype gliomas, achieving a diagnostic accuracy exceeding 97%. By leveraging accumulated metabolite biomarkers revealed by large-scale clinical investigations, this POC MS system holds strong potential for improving intraoperative diagnostics for a broad range of brain tumors as well as other types of cancers.

## Methods

### Chemicals and Materials

HPLC grade solvents (ethanol and water) were purchased from Fisher Scientific (NJ, USA). Borosilicate glass capillaries (1.5 mm o. d. and 0.86 mm i. d.) for nanoESI were purchased from Sutter Instrument (Novato, CA, USA). Borosilicate glass capillaries were washed with a solution of formic acid/ethanol/water (2/49/49, v/v/v), and then pulled to make nanoESI emitters (i.d. of 5-10 μm) using a P-1000 micropipette puller (Sutter Instrument, Novato, CA, USA).

### Acquisition of Brain Tumor Tissues

Glioma samples were collected at the Department of Neurosurgery, Huashan Hospital, following a standardized protocol approved by an independent institutional review board (IRB) at Huashan Hospital, Fudan University (KY2019-587). Informed consent was obtained from all patients. All tumor tissue samples were collected intraoperatively and preserved at -80°C within 20 min. Pathology and molecular diagnostic results were confirmed according to the WHO CNS 5 classification.

In the glioma diagnosis and margin detection study, a total of 137 samples from 109 patients were collected, comprising 49 IDH mutant samples, 74 IDH wild-type samples, and 14 normal tissues. In the intraoperative subtype classification study, 32 samples from 17 patients were collected, including 11 astrocytoma samples (6 patients), 9 oligodendroglioma samples (5 patients), 12 GBM samples (6 patients). The characteristics of patients in both cohorts are listed in Table S2 and S3.

### Intraoperative Neurosurgical Sampling

Intraoperative samples were collected during surgery in an advanced neurosurgical operating room equipped with navigation systems. For each case, a portion of the tumor tissue (approximately 0.2 cm³) was excised, placed on sterile gauze, and introduced into the miniature MS using a direct sampling cartridge. Data acquisition and analysis of intraoperative raw data were performed using PMS Client Pro software (PURSPEC Technologies, Beijing, China).

### Protocols of Direct Sampling and Ionization for Tissue Samples

The porous polymer microprobe was prepared based on our previous report^44^. The microprobe was fixed onto a 3D-printed cartridge, which consisted of a handle part and a substrate. For sampling of metabolites from tissue samples, frozen tissue samples were thawed to room temperature. The microprobe was applied onto the tissue sample by rolling on the surface or inserting into the tissue. After sampling, the probe was inserted into nanoESI emitter for elution (ethanol/water, v/v, 9/1). The ionization voltage was set at 2 kV for nanoESI in positive mode, and -2.5 kV in negative mode. For quality control, blank samples consisting of methanol were injected into the miniature MS system at the start of each daily analysis to ensure no residual compounds were detected. The standard calibration solution (PURSPEC Technologies) was used to ensure the accuracy of m/z, and a 1 ng/mL verapamil solution was used to confirm the sensitivity of the instrument. The system's reproducibility was evaluated by analyzing porcine brain extracts. Peaks at m/z 132, 146, and 281 were monitored showed relative standard deviations (RSDs) of less than 15% in intensity across parallel experiments (N = 5) (Figure S19).

### Miniature MS System

The miniature mass spectrometer was obtained from PURSPEC Technologies (Beijing, China). It has the m/z range up to 1500 and a resolving power of Δm/z 0.4 at full width at half maximum (FWHM). The size of the instrument was of 33 cm (length) × 23 cm (width) × 15 cm (height), with a weight ∼ 8 kg and power consumption ∼ 80 W. It consists of a linear ion trap mass analyzer, a compact pumping system, a direct atmospheric pressure interface (DAPI) for ion injection, and an ion source that supports nanoESI. A 24 V direct current pulse was used to control the opening and closing of the DAPI. The linear ion trap operated at a radio frequency (RF) of 1033 kHz, with resonance ejection at an alternating current (AC) frequency of 376 kHz.

The ion injection time was controlled by adjusting the delay between closing the DAPI valve and initiating the radio frequency. Following ionization, ions were injected into the ion trap at a high injection time (typically 60 ms). The segmented analysis data acquisition method is mainly realized in a mini-MS system with ion trap through the Stored Waveform Inverse Fourier Transform (SWIFT). The SWIFT technique operates by first generating a mixing frequency waveform through inverse Fourier transform algorithm. This waveform is then loaded into the ion trap to achieve selective isolation of ions within the targeted m/z range. The upper and lower cut-off frequencies of SWIFT are calculated according to the instrument parameters of the ion trap and the mass charge ratio of the ions at the beginning and end of the scanning segment. By adjusting the RF voltage, the q value of the center ion in the required isolated mass segment is 0.65, ensuring that the ions within the target range are in a stable region. When the ion trap is loaded with the SWIFT signal, except for the mass-charge ratio ions in the resonance frequency range with zero intensity component, the remaining ions will be removed from the ion trap to achieve a specific range of ion isolation. A segmented scan strategy was applied in this work, with each segment covering a mass range of 20-100 Da within the overall m/z range of 50 to 500 for analyzing metabolites in brain samples.

### Data Processing and Statistics

Data acquisition and instrument control was PMS Client Pro software (PURSPEC Technologies, Beijing, China). Data processing including peak-picking and extracting features was performed using MATLAB (v2019b). Specifically, peak intensities were extracted using a local maxima search within a ±0.3 Da window centered on each target m/z. If no local maxima were detected, the mean intensity within the window was used. For MS1 segment scans, multiple scan segments were merged based on predefined m/z boundaries to construct full-length spectra prior to feature extraction. The XGBoost classifier was trained with class-weighted optimization, followed by rigorous evaluation on a held-out test set to validate generalization performance. Relative intensity was used in data analysis. In conventional full scan acquisition, relative intensity is defined as the ratio of the area of this peak to the total area of the full-scab spectrum. In segment scan, relative intensity is defined as the ratio of the area of this peak to the total area of segment spectrum which this peak is included in. Human Metabolome Database (HMDB, www.hmdb.ca/) was used for metabolome identification. Classification models were constructed using the parameterized xgboost model and the performance of models were evaluated in the training dataset. For classification, the dataset was randomly split into training and testing subsets using a stratified 80:20 ratio to preserve class distribution. The model was trained on the training set and evaluated on the held-out test set to assess generalization performance. Besides, 5-fold cross-validation was also conducted to assess the stability and robustness. To address class imbalance in the glioma versus normal classification task, a class weighting strategy was applied within the XGBoost classifier.

Statistical analyses were performed to investigate metabolic differences between sample groups and to support model development. The input data matrix comprised metabolite features represented by relative intensities across all samples. Principal Component Analysis (PCA) was employed for unsupervised dimensionality reduction and visualization, facilitating the evaluation of overall variance structure and the natural clustering of samples. To identify metabolites that differed significantly between groups (e.g., glioma vs. normal, IDH-mutant vs. wildtype), Student's t-tests were performed using a significance threshold of p < 0.05. Differentially abundant metabolites were analyzed with volcano plots based on log₂ fold change and p-value criteria. Hierarchical clustering and heatmaps were generated to illustrate sample similarity based on discriminative metabolite profiles, applying Euclidean distance and Ward's linkage method. K-means clustering was used as an unsupervised learning approach to uncover potential sample subgroups without relying on predefined labels. All statistical analyses and visualizations were performed using the web-based platform MetaboAnalyst 5.0 (https://www.metaboanalyst.ca/).

## Supplementary Material

Supplementary figures and tables.

## Abbreviations

MS: mass spectrometry
POC: Point-of-care
2HG: 2-hydroxyglutarate
IDH: isocitrate dehydrogenase
SWIFT: stored waveform inverse Fourier transform
ROC: receiver operating characteristic (ROC) curve
AUC: area under the curve
