# Supplementary material for: Point-of-care mass spectrometry metabolomic analysis enabling intraoperative brain tumor diagnosis
Source: Theranostics. 2025 Jul 24;15(16):8137–49. doi: 10.7150/thno.113336 (PMC12374578; doi:10.7150/thno.113336)
Supplement: Supplementary file 1 — Supplementary figures and tables. [file thnov15p8137s1.pdf]

Supplementary Material

Point-of-care mass spectrometry metabolomic analysis enabling intraoperative brain tumor diagnosis

Junhan Wu[1,†], Xinqi Fang[2,3,4,5,6,†], Haoyue Zhang[1], Hao Xu[2,3,4,5,6], Peter Jih Cheng Wong[2,3,4,5,6], Ying Mao[2,3,4,5,6], Wenpeng Zhang[1*], Zheng Ouyang[1*], Wei Hua[2,3,4,5,6*]

1 State Key Laboratory of Precision Measurement Technology and Instruments, Department of Precision Instrument, Tsinghua University, Beijing 100084, China
2 Department of Neurosurgery, Huashan Hospital, Fudan University, Shanghai, 200040, China.
3 National Center for Neurological Disorders, Shanghai, 200040, China
4 Shanghai Key Laboratory of Brain Function Restoration and Neural Regeneration, Shanghai, 200040, China
5 Institute of Neurosurgery, Fudan University, Shanghai, 200040, China
6 Shanghai Clinical Medical Center of Neurosurgery, Shanghai, 200040, China

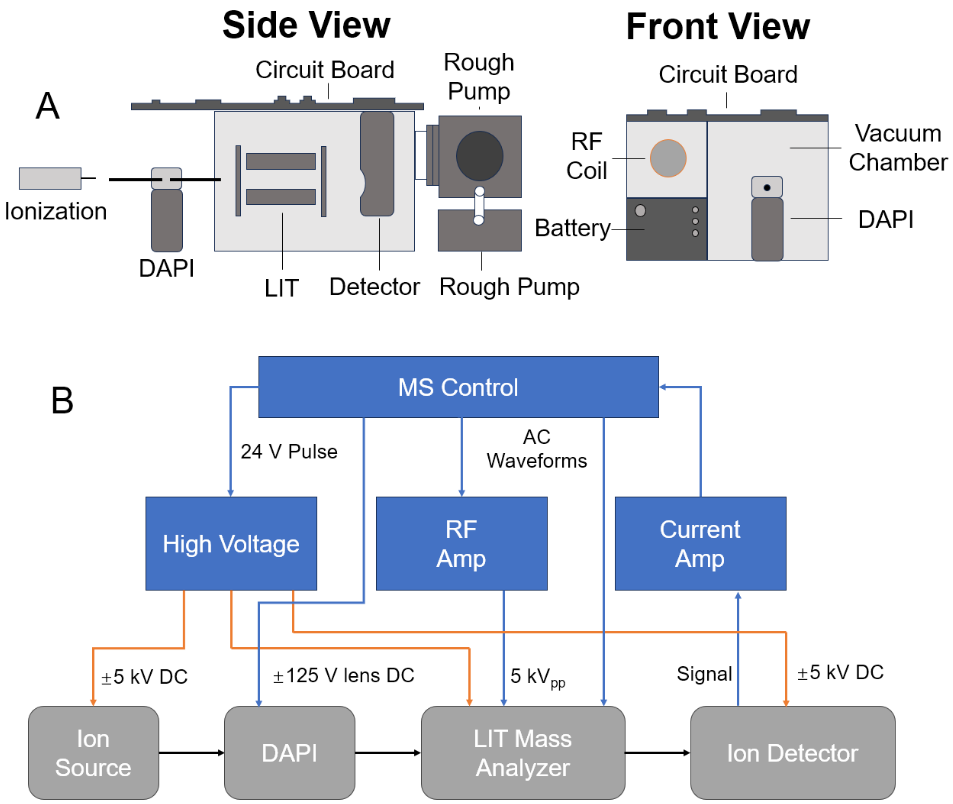

Figure S1. (A) Schematic of the miniature MS system. It consists of a discontinuous atmospheric pressure interface, a linear ion trap, electron multiplier detector and control electronics. (B) Schematic of the control electronics in the miniature MS system.

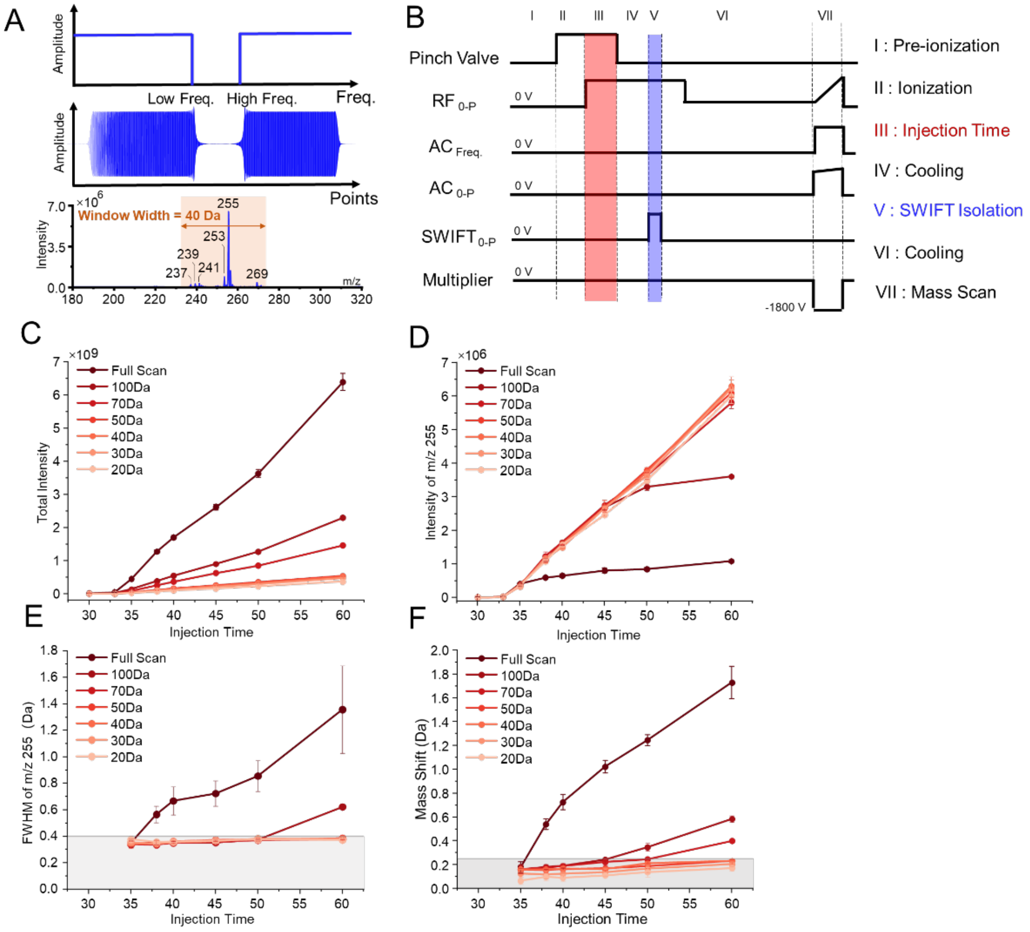

Figure S2. (A) Frequency domain graph (up) and time domain graph (middle) of SWIFT waveform for isolated a 40Da range ions centered on m/z 255. (B) Scan function for mass analysis using miniature mass spectrometer with injection control and SWIFT isolation. Effect of injection value on (C) total intensity, (D) intensity of m/z 255, (E) FWHM of m/z 255 and (F) mass shift of m/z 255 at different scan range centered on m/z 255.

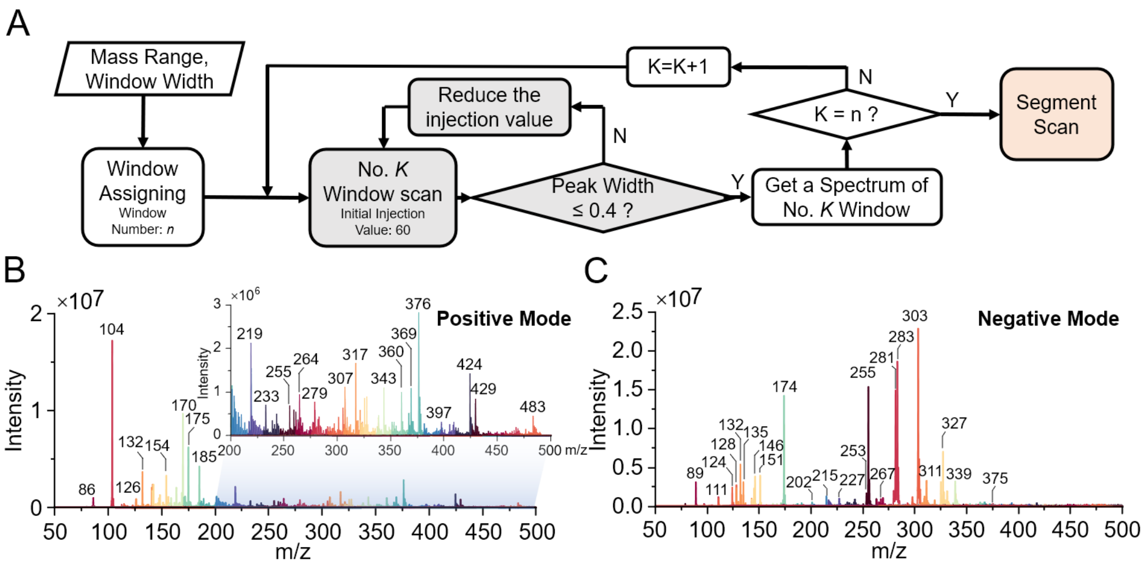

Figure S3. (A) Strategy implemented for injection control in segment scan workflow. Mass segment scan with 20 Da scan range spectra in (B) positive and (C) negative mode.

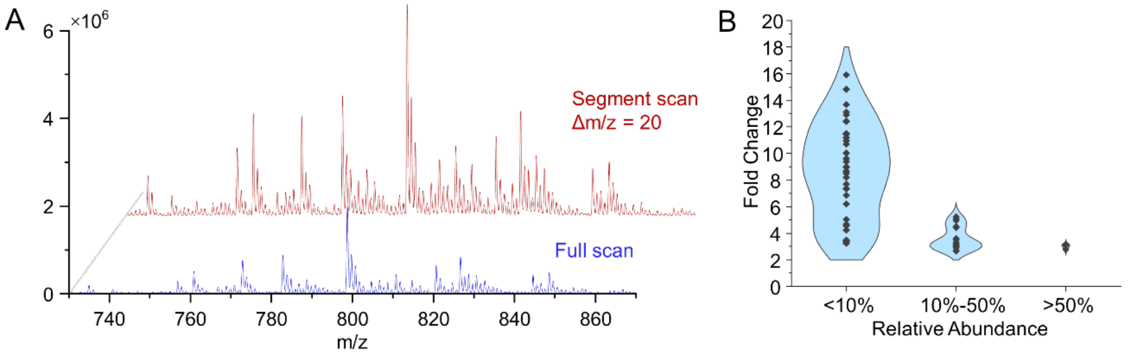

Figure S4. (A) Comparison of mass spectrum of lipid obtained by conventional full scan method and segment scan method at 20 Da window width. (B) Fold change of identified lipid peaks between segment scan and conventional full scan methods at different abundances.

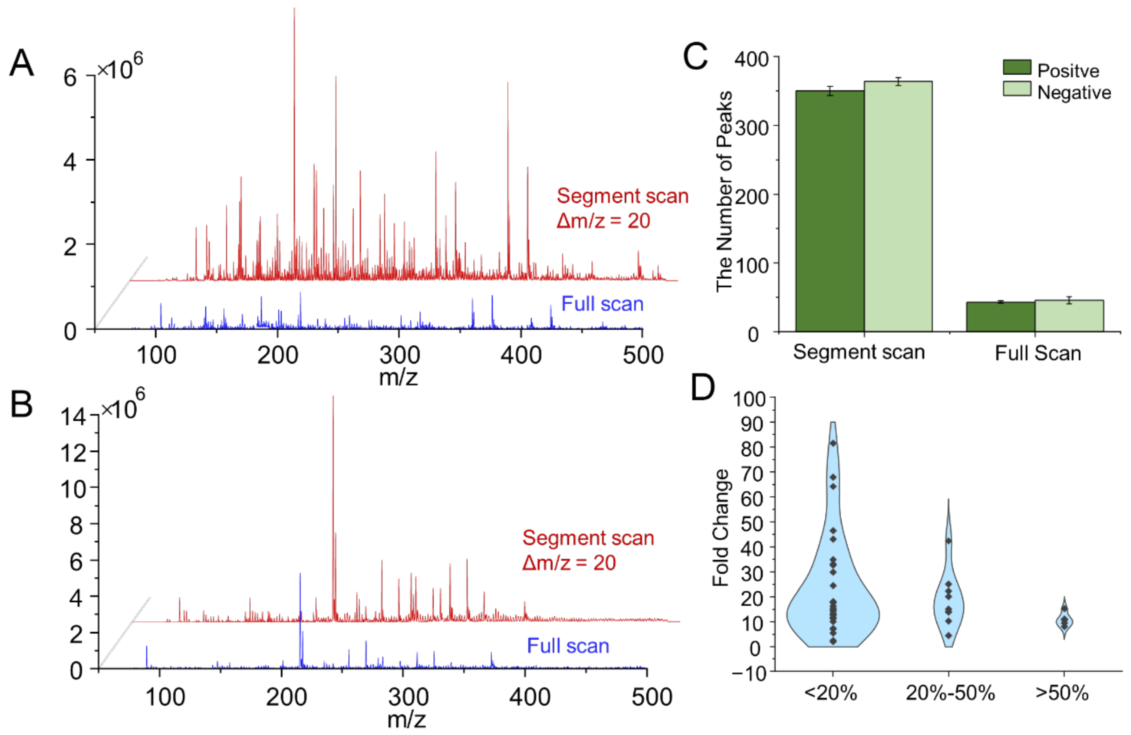

Figure S5. Comparison of mass spectrum of metabolites in whole blood obtained by conventional full scan method and segment scan method at 20 Da window width with (A) positive and (B) negative mode. (C) The number of identified peaks by conventional full scan method and segment scan method. (D) Fold change of identified lipid peaks between conventional full scan method and segment scan method at different relative abundance.

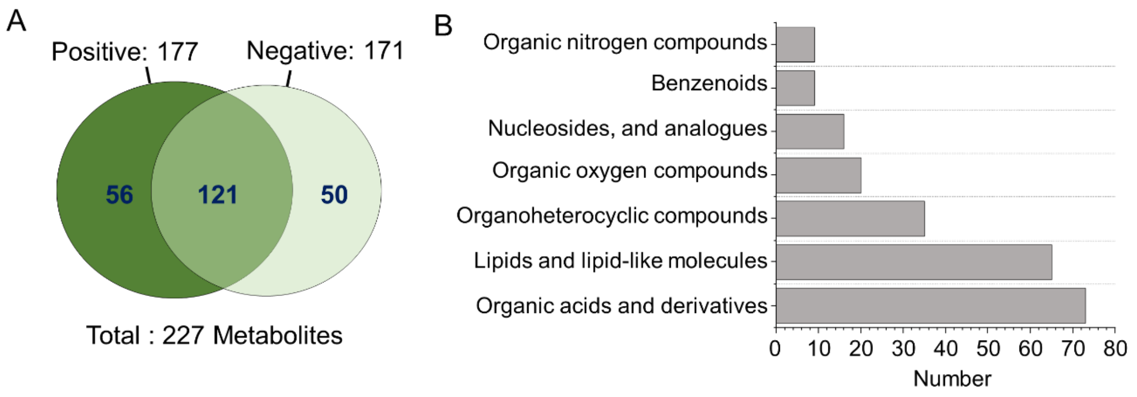

Figure S6. (A) Number of metabolites identified matching with HMDB database. (B) Number of identified metabolites in different classes.

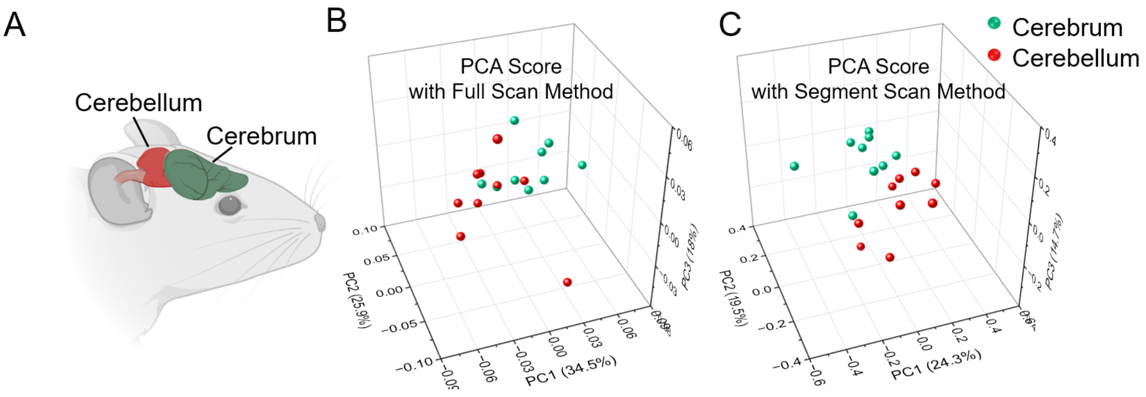

Figure S7. (A) Diagram of the location of the rat cerebrum and cerebellum. The three-dimensional PCA score map was obtained by using (B) conventional full scan and (C) segment scan methods.

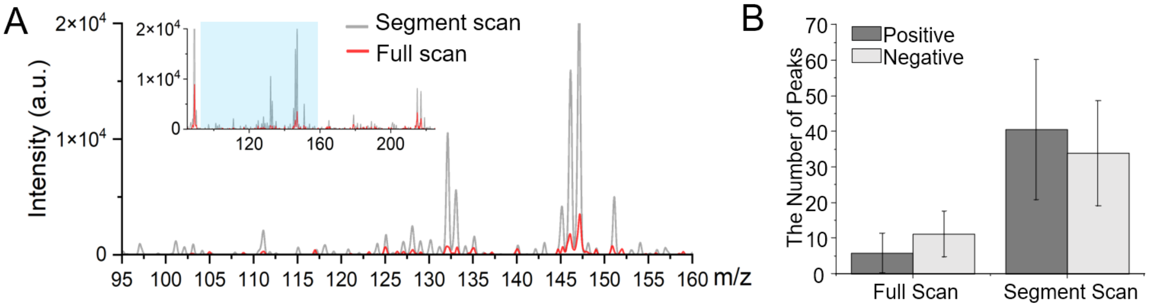

Figure S8. (A) Comparison of mass spectrum of metabolites in glioma tissue obtained by conventional full scan method and segment scan method. (B) Comparison of the number of identified peaks in all glioma samples with conventional full scan method and segment scan method at negative mode or positive mode.

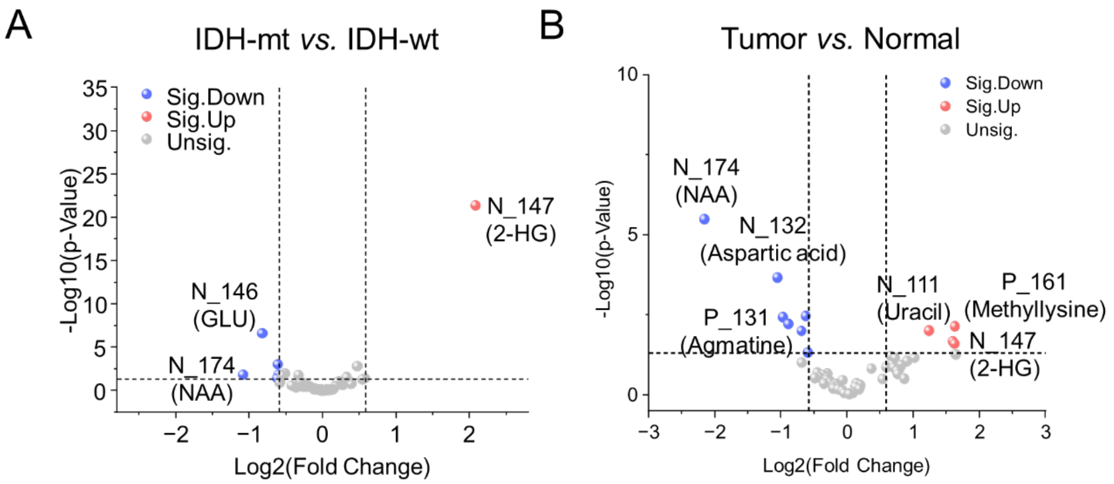

Figure S9. Volcanic plot and significantly differential metabolites that distinguish (A) IDH mutant and IDH Wildtype tissues, and (B) Glioma tumor and normal tissues using conventional full scan method.

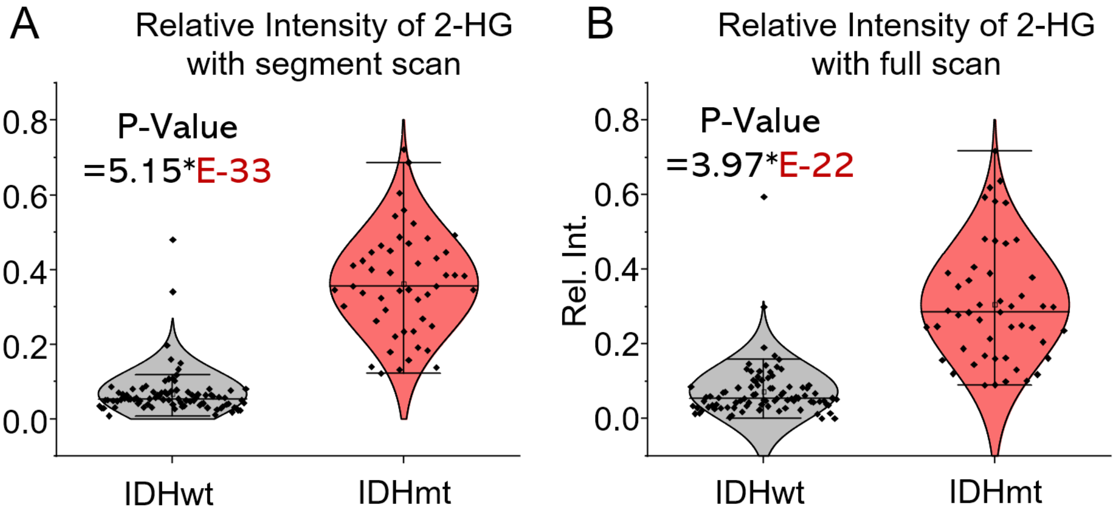

Figure S10. Comparison of the 2-HG difference in the determination of IDH mutation between (A) segment scan and (B) conventional full scan method.

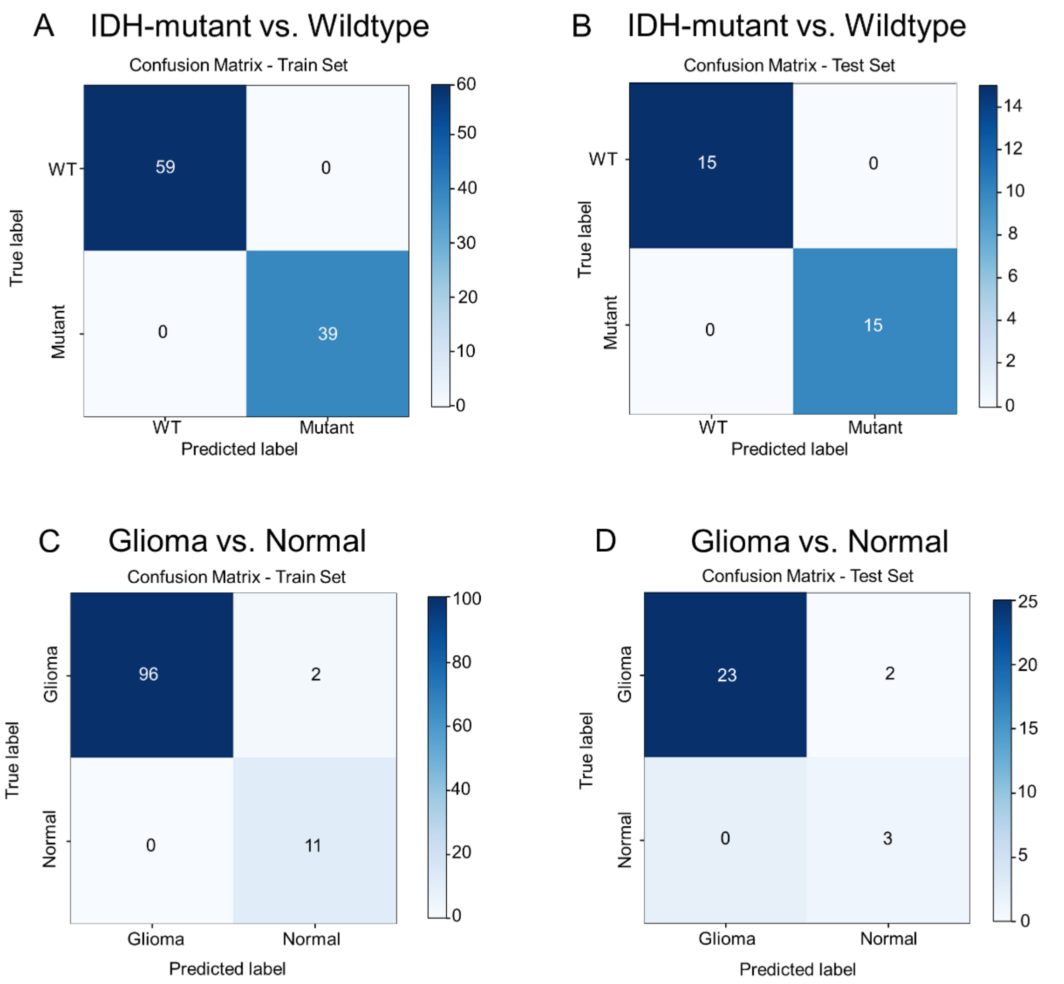

Figure S11. Confusion matrix of (A) train set and (B) test set in the classification model for IDH-mutant vs. wildtype. And (C) train set and (D) test set for glioma vs. normal.

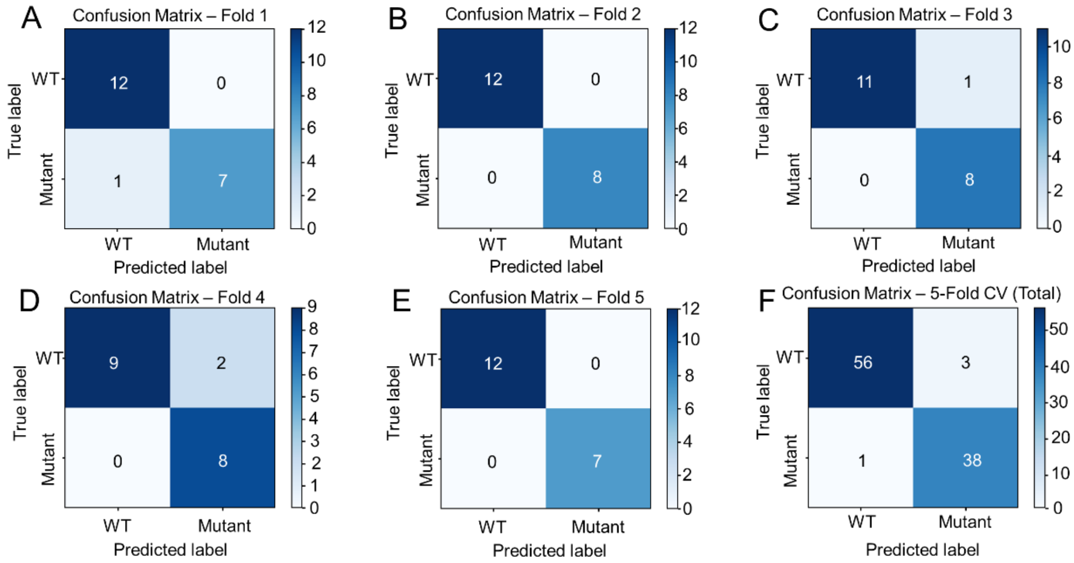

Figure S12. Confusion matrix of (A-E) Fold 1-Fold 5 and (D) average result for validation set in the 5-fold-cv for IDH-mutant vs. wildtype.

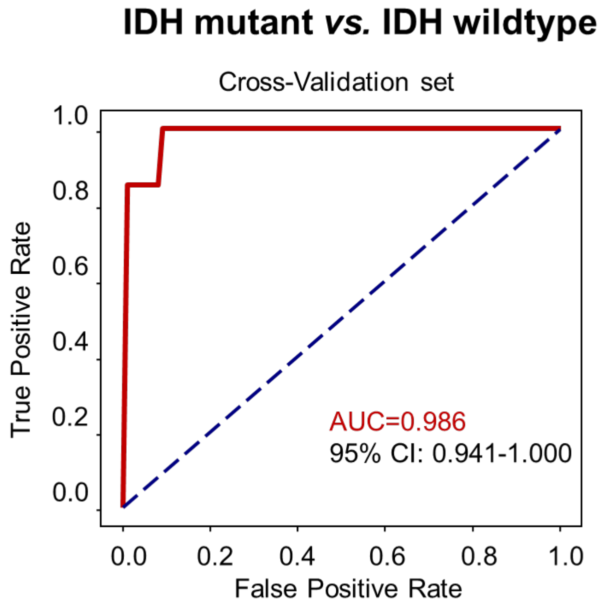

Figure S13. Mean ROC and AUC values for classification of IDH mutant versus IDH wildtype gliomas with cross-validation set.

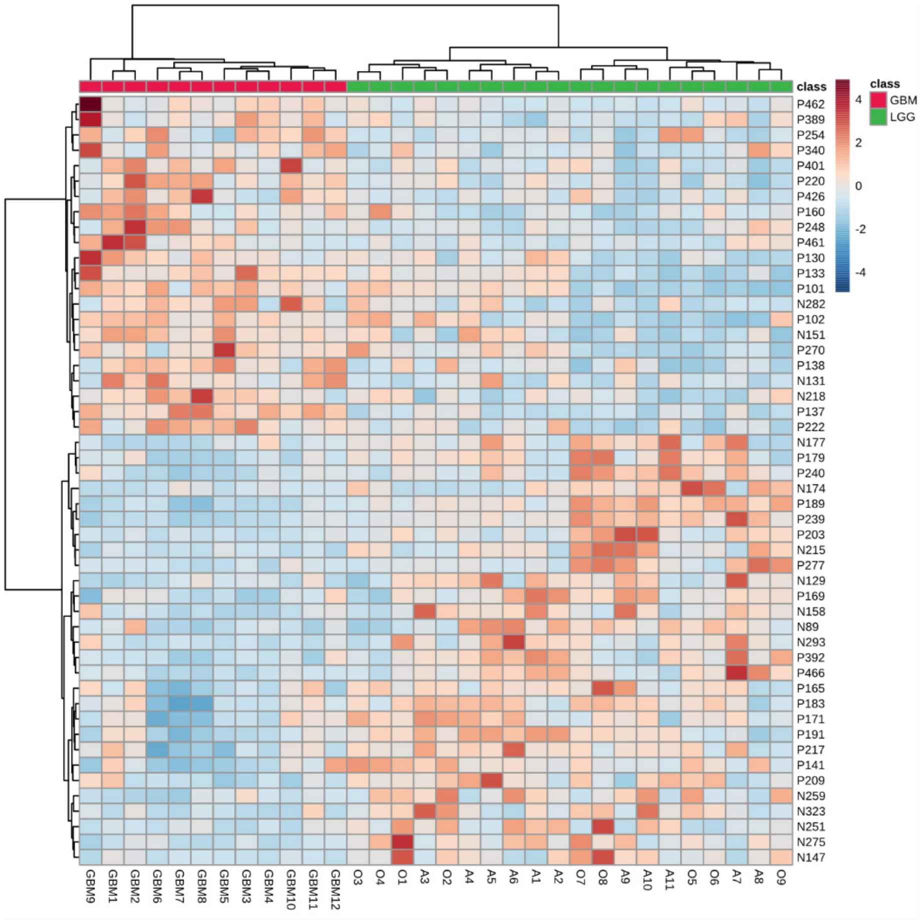

Figure S14. Hierarchical cluster analysis of GBM and LGG (Including astrocytoma and oligodendroglioma) using metabolites features.

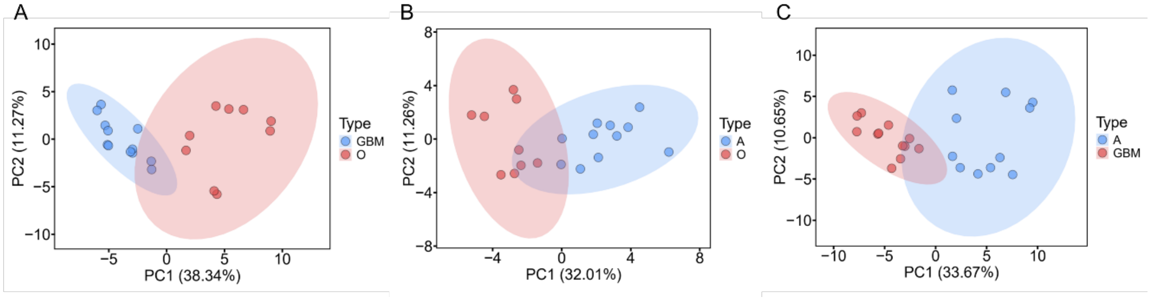

Figure S15. Principal component analysis (PCA) of (A) GBM vs. oligodendroglioma, (B) astrocytoma vs. oligodendroglioma, (C) GBM vs. astrocytoma using metabolomic features.

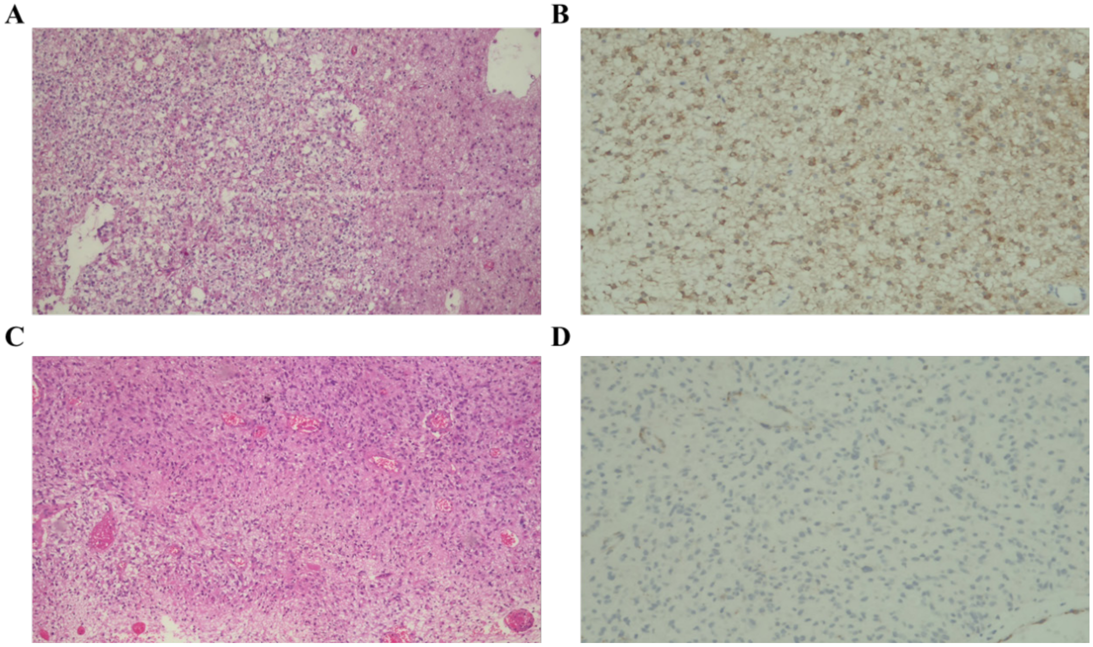

Figure S16. Postoperative histopathological validation of glioma subtypes and IDH status for the cases shown in Figure 6. (A, B) Case 1 (left frontal glioma): (A) Hematoxylin and eosin (HE) staining of the tumor core; (B) positive immunostaining for IDH R132H confirms IDH-mutant status. (C, D) Case 2 (right parietal tumor): (C) HE staining of the tumor core; (D) negative immunostaining for IDH R132H confirms IDH-wildtype status.

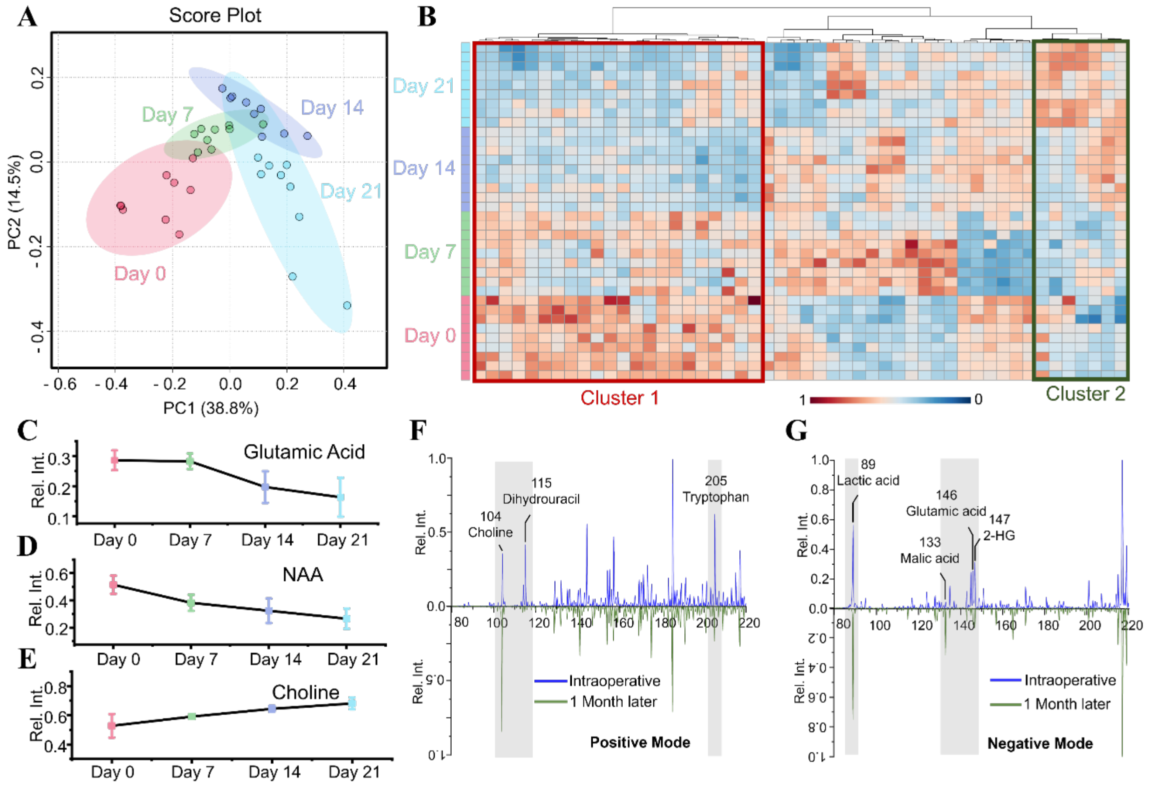

Figure S17. (A) Principal component analysis (PCA) of a same rat brain tissue in different weeks using metabolites features in positive and negative ion mode data. (B) Hierarchical cluster analysis of brain tissue in different weeks using metabolites features. Trend of relative intensity of (C) glutamic acid, (D) NAA and (E) choline in rat brain over 21 Days. Comparison of spectrum of a glioma tissue in (F) positive and (G) negative mode between intraoperative and 1 month ago analysis.

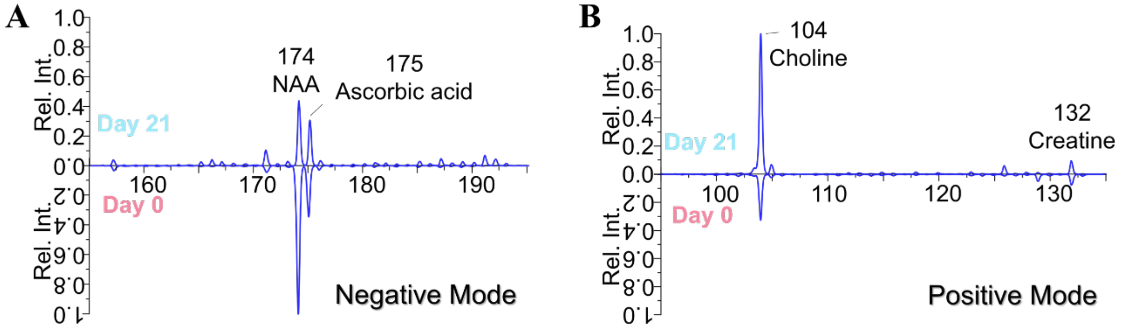

Figure S18. Comparison of spectrum of (A) NAA and (B) choline between analysis of Day 0 and Day 21 for glioma tissues.

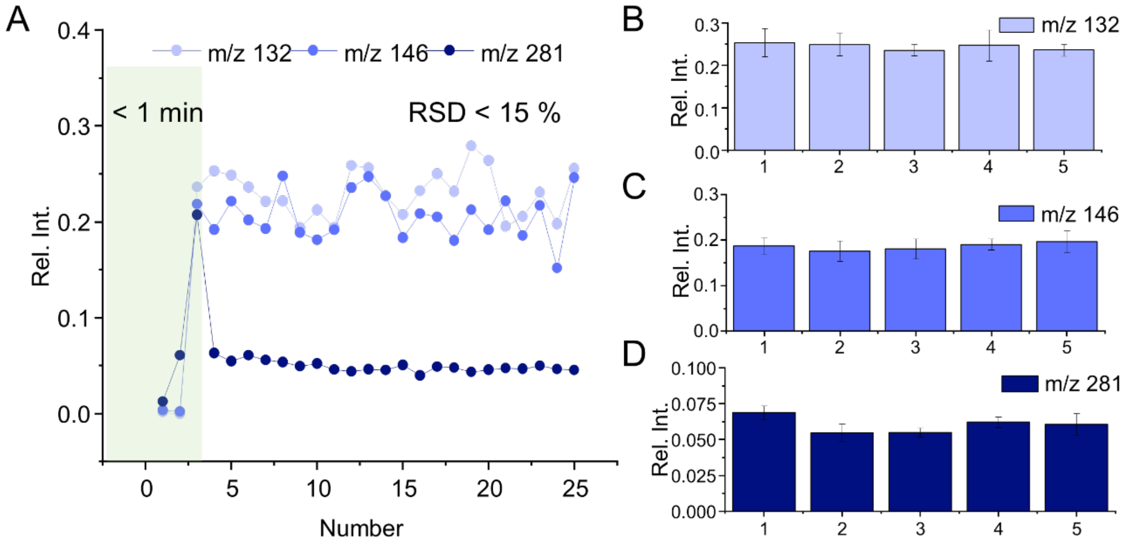

Figure S19. (A) Comparison of the results of multiple continuous injections with the same probe. Comparison of the consistency of results from five different probes with (B) m/z 132, (C) m/z 146 and (D) m/z 281.

Table S1 Widow distribution of different spectra stitching width and the number of times within the range of m/z 70- m/z 500 with each spectra stitching strategy.

Single Scan Range	Overlap Range	Number of Scan
20	5	28
30	10	21
40	10	14
50	10	11
70	10	7
100	10	5
Full Scan	0	1

Table S2: The characteristics of patients in the glioma diagnosis cohort subjected to sequencing and pathological evaluation

Subject#	Gender	Age	Diagnosis	Grade	biopsy# (subject)	Biopsy# (overall)	Tissue	IHC	PCRseq
1	Male	16	Glioblastoma	4	1	1	Tumor tissue	-	Wildtype
2	Female	32	Astrocytoma	2	1	2	Tumor tissue	+	R132H
3	Female	44	Glioblastoma	4	1	3	Tumor tissue	-	Wildtype
4	Male	60	Glioblastoma	4	1	4	Tumor tissue	-	Wildtype
5	Male	66	Glioblastoma	4	1	5	Tumor tissue	-	Wildtype
6	Female	56	Glioblastoma	4	1	6	Tumor tissue	-	Wildtype
7	Female	58	Astrocytoma	2	1	7	Tumor tissue	-	Wildtype
	Female	58	Astrocytoma	2	2	8	Tumor tissue	-	Wildtype
	Female	58	Astrocytoma	2	3	9	Tumor tissue	-	Wildtype
8	Male	55	Astrocytoma	3	1	10	Tumor tissue	-	Wildtype
9	Male	52	Oligodendroglioma	2	1	11	Tumor tissue	+	R132H
	Male	52	Oligodendroglioma	2	2	12	Tumor tissue	+	R132H
10	Male	30	Astrocytoma	4	1	13	Tumor tissue	+	R132H
11	Female	28	Glioblastoma	4	1	14	Tumor tissue	-	Wildtype
12	Female	25	Astrocytoma	2	1	15	Tumor tissue	-	Wildtype
	Female	25	Astrocytoma	2	2	16	Tumor tissue	-	Wildtype
13	Male	34	Glioblastoma	4	1	17	Tumor tissue	-	Wildtype
14	Male	60	Glioblastoma	4	1	18	Tumor tissue	-	Wildtype
15	Male	45	Astrocytoma	3	1	19	Tumor tissue	-	Wildtype
16	Male	42	Astrocytoma	2	1	20	Tumor tissue	+	R132H
	Male	42	Astrocytoma	2	2	21	Tumor tissue	+	R132H
17	Female	35	Oligodendroglioma	3	1	22	Tumor tissue	+	R132H
18	Female	50	Oligodendroglioma	3	1	23	Tumor tissue	+	R132H
19	Female	28	Oligodendroglioma	3	1	24	Tumor tissue	+-	R132H
20	Female	69	Astrocytoma	3	1	25	Tumor tissue	-	Wildtype
21	Male	70	Glioblastoma	4	1	26	Tumor tissue	-+	Wildtype
	Male	70	Glioblastoma	4	2	27	Tumor tissue	-+	Wildtype
22	Female	34	Astrocytoma	2	3	28	Tumor tissue	+	R132H
23	Male	44	Astrocytoma	2	1	29	Tumor tissue	-	R132H
24	Male	66	Glioblastoma	4	1	30	Tumor tissue	-	Wildtype
25	Male	80	Glioblastoma	4	1	31	Tumor tissue	-	Wildtype
26	Female	30	Astrocytoma	2	1	32	Tumor tissue	-	Wildtype
27	Male	22	Astrocytoma	3	1	33	Tumor tissue	+	R132H
28	Male	47	Glioblastoma	4	1	34	Tumor tissue	-	Wildtype
29	Male	64	Glioblastoma	4	1	35	Tumor tissue	-	Wildtype

30	Female	47	Glioblastoma	4	1	36	Tumor tissue	-	Wildtype
31	Male	50	Glioblastoma	4	1	37	Tumor tissue	-	Wildtype
32	Female	23	Astrocytoma	2	1	38	Tumor tissue	-	Wildtype
33	Male	47	Glioblastoma	4	1	39	Tumor tissue	-	Wildtype
34	Male	45	Glioblastoma	4	1	40	Tumor tissue	-	Wildtype
35	Male	43	Astrocytoma	2	1	41	Tumor tissue	+	R132H
36	Female	53	Astrocytoma	2	1	42	Tumor tissue	-	R132H
37	Female	32	Astrocytoma	3	1	43	Tumor tissue	+	R132H
38	Male	68	Glioblastoma	4	1	44	Tumor tissue	-	Wildtype
39	Female	65	Glioblastoma	4	1	45	Tumor tissue	-	Wildtype
40	Male	55	Glioblastoma	4	1	46	Tumor tissue	-	Wildtype
41	Female	47	Pilocyticastrocytoma	1	1	47	Tumor tissue	-	Wildtype
42	Male	24	Astrocytoma	2	1	48	Tumor tissue	-	Wildtype
43	Male	42	Glioblastoma	4	1	49	Tumor tissue	-	Wildtype
44	Male	59	Glioblastoma	4	1	50	Tumor tissue	-	Wildtype
45	Male	36	Oligodendroglioma	2	1	51	Tumor tissue	-+	R132H
46	Male	33	Astrocytoma	2	1	52	Tumor tissue	+-	Wildtype
	Male	33	Astrocytoma	2	2	53	Tumor tissue	+-	Wildtype
47	Female	63	Glioblastoma	4	1	54	Tumor tissue	-	Wildtype
48	Male	15	Glioblastoma	4	1	55	Tumor tissue	-	Wildtype
49	Male	65	Glioblastoma	4	1	56	Tumor tissue	-	Wildtype
50	Male	41	Astrocytoma	2	1	57	Tumor tissue	-	Wildtype
51	Female	60	Glioblastoma	4	1	58	Tumor tissue	-	Wildtype
52	Female	60	Astrocytoma	3	1	59	Tumor tissue	-	Wildtype
53	Male	53	Astrocytoma	3	1	60	Tumor tissue	-	Wildtype
54	Female	55	Oligodendroglioma	2	1	61	Tumor tissue	+	R132H
55	Female	50	Glioblastoma	4	1	62	Tumor tissue	-	Wildtype
56	Female	32	Astrocytoma	4	1	63	Tumor tissue	+	R132H
57	Male	47	Oligodendroglioma	3	1	64	Tumor tissue	+	R132H
58	Male	47	Astrocytoma	4	1	65	Tumor tissue	+	R132H
59	Female	45	Oligodendroglioma	2	1	66	Tumor tissue	+	R132H
60	Male	36	Astrocytoma	2	1	67	Tumor tissue	+	R132H
	Male	36	Astrocytoma	2	2	68	Tumor tissue	+	R132H
	Male	36	Astrocytoma	2	3	69	Tumor tissue	+	R132H
61	Male	48	Astrocytoma	2	1	70	Tumor tissue	-	R172K
62	Female	22	Pilocyticastrocytoma	1	1	71	Tumor tissue	-	Wildtype
63	Female	34	Astrocytoma	2	1	72	Tumor tissue	+	R132H
	Female	34	Astrocytoma	2	2	73	Tumor tissue	+	R132H
64	Male	56	Glioblastoma	4	1	74	Tumor tissue	-	Wildtype
65	Male	25	Astrocytoma	2	1	75	Tumor tissue	-	R172K

66	Female	34	Astrocytoma	4	1	76	Tumor tissue	+	R132H
	Female	34	Astrocytoma	4	2	77	Tumor tissue	+	R132H
67	Female	32	Oligodendroglioma	3	1	78	Tumor tissue	+	R132H
68	Male	61	Glioblastoma	4	1	79	Tumor tissue	-	Wildtype
69	Female	77	Glioblastoma	4	1	80	Tumor tissue	-	Wildtype
70	Male	38	Glioblastoma	4	1	81	Tumor tissue	-	Wildtype
71	Male	50	Oligodendroglioma	2	1	82	Tumor tissue	+	R132H
	Male	50	Oligodendroglioma	2	2	83	Tumor tissue	+	R132H
72	Female	43	Astrocytoma	2	1	84	Tumor tissue	+	R132H
73	Male	48	Glioblastoma	4	1	85	Tumor tissue	-	Wildtype
74	Male	59	Oligodendroglioma	3	1	86	Tumor tissue	+	R132H
	Male	57	Oligodendroglioma	2	2	87	Tumor tissue	+	R132H
75	Male	53	Oligodendroglioma	2	1	88	Tumor tissue	+	R132H
76	Male	43	Oligodendroglioma	2	1	89	Tumor tissue	-	R132H
77	Female	74	Glioblastoma	4	1	90	Tumor tissue	-	Wildtype
78	Male	41	Astrocytoma	2	1	91	Tumor tissue	-	R132H
79	Male	44	Oligodendroglioma	3	1	92	Tumor tissue	-+	R132H
80	Male	45	Glioblastoma	4	1	93	Tumor tissue	-	Wildtype
81	Female	33	Glioblastoma	4	1	94	Tumor tissue	-	Wildtype
82	Male	62	Glioblastoma	4	1	95	Tumor tissue	-	Wildtype
83	Female	20	Glioblastoma	4	1	96	Tumor tissue	-	Wildtype
	Female	20	Glioblastoma	4	2	97	Tumor tissue	-	Wildtype
	Female	20	Glioblastoma	4	3	98	Tumor tissue	-	Wildtype
84	Male	15	Astrocytoma	3	1	99	Tumor tissue	-	Wildtype
85	Male	33	Glioblastoma	4	1	100	Tumor tissue	-	Wildtype
86	Female	38	Oligodendroglioma	2	1	101	Tumor tissue	+	R132H
87	Female	54	Oligodendroglioma	3	1	102	Tumor tissue	+	R132H
88	Male	36	Oligodendroglioma	3	1	103	Tumor tissue	+	R132H
89	Female	44	Astrocytoma	3	1	104	Tumor tissue	-+	R132H
90	Male	54	Glioblastoma	4	1	105	Tumor tissue	-	Wildtype
91	Male	51	Astrocytoma	3	1	106	Tumor tissue	+	R132H
92	Female	26	Oligodendroglioma	2	1	107	Tumor tissue	+	R132H
93	Male	37	Oligodendroglioma	2	1	108	Tumor tissue	+	R132H
94	Male	39	Glioblastoma	4	1	109	Tumor tissue	-	Wildtype
95	Male	50	Glioblastoma	4	1	110	Tumor tissue	-	Wildtype
96	Male	39	Glioblastoma	4	1	111	Tumor tissue	-	Wildtype
97	Male	68	Glioblastoma	4	1	112	Tumor tissue	-	Wildtype
	Male	68	Glioblastoma	4	2	113	Tumor tissue	-	Wildtype
98	Male	50	Astrocytoma	2	1	114	Tumor tissue	-+	R132H
99	Female	61	Glioblastoma	4	1	115	Tumor tissue	-	Wildtype

	Female	61	Glioblastoma	4	2	116	Tumor tissue	-	Wildtype
	Female	61	Glioblastoma	4	3	117	Tumor tissue	-	Wildtype
	Female	61	Glioblastoma	4	4	118	Tumor tissue	-	Wildtype
	Female	61	Glioblastoma	4	5	119	Para-tumor brain tissue	-	Wildtype
100	Male	47	Oligodendroglioma	3	1	120	Tumor tissue	-	R132H
101	Male	64	Glioblastoma	4	1	121	Tumor tissue	-	Wildtype
102	Female	69	Glioblastoma	4	1	122	Tumor tissue	-	Wildtype
103	Male	64	Astrocytoma	3	1	123	Tumor tissue	-	Wildtype
104	Male	42	Glioblastoma	4	1	124	Tumor tissue	-	Wildtype
105	Female	56	Meningioma	1	1	125	Normal brain tissue	-	Wildtype
106	Female	35	Meningioma	1	1	126	Normal brain tissue	-	Wildtype
	Female	35	Meningioma	1	2	127	Normal brain tissue	-	Wildtype
	Female	35	Meningioma	1	3	128	Normal brain tissue	-	Wildtype
	Female	35	Meningioma	1	4	129	Normal brain tissue	-	Wildtype
107	Female	56	Neurilemmoma	1	1	130	Normal brain tissue	-	Wildtype
	Female	56	Neurilemmoma	1	2	131	Normal brain tissue	-	Wildtype
	Female	56	Neurilemmoma	1	3	132	Normal brain tissue	-	Wildtype
108	Male	57	Astrocytoma	2	1	133	Normal brain tissue	-	Wildtype
	Male	57	Astrocytoma	2	2	134	Para-tumor brain tissue	-	Wildtype
	Male	57	Astrocytoma	2	3	135	Para-tumor brain tissue	-	Wildtype
109	Female	53	Meningioma	1	1	136	Normal brain tissue	-	Wildtype
	Female	53	Meningioma	1	2	137	Normal brain tissue	-	Wildtype

Table S3: The characteristics of patients in the intraoperative subtype classification cohort subjected to sequencing and pathological evaluation.

Subject#	Gender	Age	Diagnosis	Grade	IHC	PCRseq
1	Male	43	Astrocytoma	3	+	R132H
2	Male	53	Astrocytoma	3	+	R132H
3	Male	43	Astrocytoma	2	+	R132H
4	Female	37	Astrocytoma	3	+	R132H
5	Male	52	Astrocytoma	2	+	R132H
6	Male	57	Astrocytoma	2	+	R132H
7	Male	58	Glioblastoma	4	-	Wildtype
8	Male	66	Glioblastoma	4	-	Wildtype
9	Female	67	Glioblastoma	4	-	Wildtype
10	Female	47	Glioblastoma	4	-	Wildtype
11	Female	72	Glioblastoma	4	-	Wildtype
12	Female	64	Glioblastoma	4	-	Wildtype
13	Female	40	Oligodendroglioma	3	+	R132H
14	Female	36	Oligodendroglioma	2	+	R132H
15	Male	32	Oligodendroglioma	3	+	R132H
16	Male	42	Oligodendroglioma	3	+	R132H
17	Male	54	Oligodendroglioma	3	+	R132H

Table S4: Evaluation results of classification models for IDH-mutant vs. wildtype and glioma vs. normal.

	**IDH-mutant vs. Wildtype**		**Glioma vs. Normal**	
	Train Set	Test Set	Train Set	Test Set
Accuracy	1.000	1.000	0.982	0.929
Error Rate	0.000	0.000	0.018	0.071
Sensitivity (Recall)	1.000	1.000	1.000	1.000
Specificity	1.000	1.000	0.980	0.920
Precision	1.000	1.000	0.846	0.600
F1 Score	1.000	1.000	0.917	0.750
AUC	1.000 (CI: 1.000 - 1.000)	1.000 (CI: 1.000 - 1.000)	0.998 (CI: 0.992 - 1.000)	0.960 (CI: 0.852 - 1.000)

Table S5: Evaluation results of 5-fold-cv for IDH-mutant vs. wildtype.

	**IDH-mutant vs. Wildtype （5-fold-cross validation）**					
	Fold 1	Fold 2	Fold 3	Fold 4	Fold 5	Mean
Accuracy	0.850	0.900	1.000	1.000	0.947	0.959
Sensitivity (Recall)	0.875	1.000	1.000	1.000	1.000	0.975
Specificity	1.000	1.000	0.917	0.818	1.000	0.946
Precision	1.000	1.000	0.889	0.800	1.000	0.937
F1 Score	0.933	1.000	0.941	0.889	1.000	0.952
AUC	0.990 (CI: 0.939-1.000)	1.000 (CI: 1.000 - 1.000)	0.979 (CI: 0.901-1.000)	0.966 (CI: 0.864-1.000)	1.000 (CI: 1.000-1.000)	0.986 (CI: 0.941-1.000)
